# *Fus*-depleted oligodendrocytes reduce neuronal damage and Alzheimer’s disease progression in the *App*^NL-G-F^ mouse

**DOI:** 10.1093/brain/awag234

**Published:** 2026-07-06

**Authors:** Te-Hsuan Tung, Sudhagar Babu, Xiangting Tang, Alec L. Sciutto, Micah Romer, Prathima Racha, Rong Xu, Victoria Fiesler, Adrita Saha, Teresa Thai, Takashi Saito, Takaomi C. Saido, Yongxin Zhao, Judith B. Grinspan, Hansruedi Mathys, Takashi D. Y. Kozai, Franca Cambi

**Affiliations:** 1 Pittsburgh Institute for Neurodegenerative Diseases, School of Medicine, University of Pittsburgh, Pittsburgh, PA 15206, USA; 2 Department of Neurobiology, School of Medicine, University of Pittsburgh, Pittsburgh, PA 15206, USA; 3 Children Hospital of Philadelphia, Perelman School of Medicine at the University of Pennsylvania, Philadelphia, PA 19104, USA; 4 Department of Biological Sciences, Carnegie Mellon University, Pittsburgh, PA,15213 USA; 5 Department of Bioengineering, University of Pittsburgh, Pittsburgh, PA 15213, USA; 6 Center for the Neural Basis of Cognition, University of Pittsburgh and Carnegie Mellon University, Pittsburgh, PA, 15213, USA; 7 Department of Neurocognitive Science, Institute of Brain Science, Nagoya City University, Nagoya, Aichi, 467-8601, Japan; 8 Department of Neuropathology, Graduate School of Medicine, The University of Tokyo, Tokyo, 113-0033, Japan; 9 RIKEN Brain Science Institute, Wako, Saitama, 351-0198, Japan; 10 Carnegie Mellon Neuroscience Institute, Carnegie Mellon University, Pittsburgh, PA, 15213, USA; 11 McGowan Institute for Regenerative Medicine, University of Pittsburgh, Pittsburgh, PA, 15219, USA; 12 Center for Neuroscience, University of Pittsburgh, Pittsburgh, PA, 15260, USA; 13 VA Pittsburgh Healthcare System, Pittsburgh, PA, 15240, USA

**Keywords:** mitochondria, neuroprotection, neuroinflammation, lipid metabolism, transcriptomics, microglia

## Abstract

Alzheimer’s Disease (AD) is an age-dependent neurodegenerative disorder and represents the most common type of dementia, increasing in incidence at an alarming rate in the aging population. The hallmarks of the disease are amyloid plaque accumulation, microglia and astrocyte activation, and loss of presynaptic structure leading to cognitive decline. Recently, oligodendrocyte (OL) and myelin abnormalities have emerged as important contributors to the pathogenesis of AD. In normal brain homeostatic conditions, OL maintain neuronal health through myelin axon interactions and by supplying neurotrophic and metabolic support. How strengthening OL function may support neuronal health in AD neurodegeneration remains to be fully characterized and represents a gap in knowledge and a missed therapeutic opportunity.

This study sought to examine how myelin and OL may improve neuronal deficits associated with AD. We have generated a novel mouse model (AD/cKO) by crossing the App^NL-G-F^ mouse, an established AD model, which carries three human AD mutations in the mouse *App* gene, with the *Fus^OL^*cKO whose OL depleted of *Fus* (Fused in Sarcoma) produce thicker myelin associated with greater cholesterol biosynthesis. We evaluated spatial memory function with standardized cognitive testing. We evaluated microglia density and state, astrocytic activation and toxic phenotype, myelin density, cholesterol content, amyloid plaque burden, presynaptic structures, and neuronal hypoxic and oxidative damage in the hippocampus and cortex. We characterized the transcriptome of AD/cKO hippocampal OL compared to AD by using single-cell transcriptomic studies.

Spatial working memory was fully preserved in the aged AD/cKO mouse relative to the AD mouse. This outcome was associated with reduced neuronal oxidative damage, preserved presynaptic structures at the amyloid plaque niches, and a shift in microglia state at the niches in both hippocampus and cortex. In contrast, amyloid plaque burden and microglia density were decreased in the hippocampus but not in cortex, uncoupling the neuronal and microglia effects from the amyloid burden. *Fus* dependent myelin increase was present in both hippocampus and cortex. Single-cell transcriptomics of AD/cKO hippocampal OL revealed upregulation of energy metabolism and antioxidant genes, suggesting a role of OL enhanced energy metabolism in mediating protection of neurons and affecting microglia state in AD pathology.

This work provides new insight into how oligodendrocytes may protect neurons in AD, communicate with other glial cellular players, and point to potential targets for disease intervention aimed at slowing AD progression.

## Introduction

Alzheimer’s Disease (AD) is an age-dependent neurodegenerative disorder and represents the most common type of dementia in the aging population. White matter abnormalities were identified by Magnetic Resonance Imaging in patients with amnestic Mild Cognitive Impairment and prodromal AD before severe cognitive deficits manifest.^1–6^ Gene expression profiling in AD brains revealed that oligodendrocytes (OL) are severely impacted early in the AD course and throughout disease progression^7^ and suggest that disruption of OL integrity is a critical mechanism in AD pathogenesis.

OL the myelin producing cells of the central nervous system support neural network integrity and function by facilitating saltatory conduction through myelin deposition around axons, providing metabolic and trophic support to neurons^8^, maintaining axon integrity and function^9–13^ and enhancing synapse formation.^14^ Cholesterol, the major lipid constituent of myelin, is reduced in the white matter of AD brains^15^ and AD risk variants, Apolipoprotein E and Clusterin, reduce myelin integrity by affecting cholesterol transport in OL, ^16–18,19,20^ demonstrating that OL are more vulnerable than other brain cells to genetic variations associated with AD. Loss of critical oligodendrocytic functions has emerged as an important contributor to the pathobiology of AD.

The role of OL and myelin has been examined in AD mouse models by either reducing myelin stability or enhancing oligodendrogenesis. Myelin loss worsened pathology by interfering with microglia degradation of Amyloid β (Aβ) associated with a shift to an inflammatory microglia state without clear worsening of cognitive decline.^21^ Enhancing oligodendrogenesis reduced AD-related cognitive decline without correcting pre-existing myelin degradation, affecting plaque burden and microglia density.^22^ Finally, a selective vulnerability of myelin-axon interactions without loss of compact myelin was uncovered in both AD brains and mouse models.^23^ These studies highlight a complex relationship between OL, myelin, neurons, amyloid deposition and microglia in the context of AD pathology. A gap in current knowledge is whether strong pre-existing myelin and oligodendrocytic metabolic capacity maintain neuronal homeostasis in AD.

We have addressed this question by leveraging the *Fus*^OL^cKO mouse that carries conditional depletion of Fused in Sarcoma (*Fus*) in OL.^24^ This mouse has greater myelin thickness and higher percentage of myelinated small caliber axons driven by enhanced cholesterol biosynthesis. Chronic electrophysiological recording *in vivo* in the *Fus*^OL^cKO visual cortex and the underlying hippocampus revealed sustained visually evoked single-unit detectability and firing rate selectively in the CA1 subregion relative to wild-type littermates.^25^ The data indicates that *Fus* depleted OL support circuit integrity in the hippocampus, a critical component of the memory loop.

Motivated by the in vivo electrophysiological results, we reasoned that *Fus* depleted OL may strengthen hippocampal function and lead to better memory preservation in AD. We have generated a novel AD mouse model by crossing the *App*^NL-G-F^ mouse, a model of prodromal AD,^26^ with the *Fus*^OL^cKO and examined AD disease progression in the hippocampus and cortex of the *App*^NL-G-F^/ *Fus*^OL^cKO (AD/cKO) and *App*^NL-G-F^ (AD). Spatial working memory was preserved in the aged AD/cKO mouse relative to the AD mouse. This outcome was associated with reduced cellular and neuronal oxidative damage, preserved presynaptic structures, and a shift in microglia state at the plaque niches in both hippocampus and cortex. In contrast, plaque burden and microglia density were decreased in the hippocampus but not in cortex, uncoupling the neuronal and microglia effects from the amyloid burden. *Fus* dependent myelin increase was present in both hippocampus and cortex. Single cell transcriptomics of AD/cKO hippocampal OL revealed upregulation of energy metabolism and antioxidant genes, suggesting a role of OL enhanced energy metabolism in protecting neurons and affecting microglia state in AD pathology.

## Materials and methods

Detailed Methods are in Supplementary Material.

### Mouse Behavioural testing

Mice were bred and housed in a barrier facility with a 12-hour light/dark cycle, food and water ad libitum. All efforts were made to minimize animal suffering and the number used. The use of animals was approved by the Institutional Animal Care and Use Committees of the University of Pittsburgh (Animal Welfare Assurance number D16-00118) under protocol # 24034725.

#### Novel Spatial Recognition

Short-term recognition memory was assessed with the novel spatial recognition test which is similar to the novel object recognition but replaces the use of objects with visual cues, using established protocols ^27,28^ The time spent in each arm and the number of entries into each arm were tracked (EthoVision XT 9.0, Noldus Information Technology, Wageningen, Netherlands). The time spent in each arm was calculated as:

(1) $$Duration\;in\;arm\;X\;(\%)=\frac{Time\;spent\;in\;arm\;X}{Sum\;of\;time\;spent\;in\;all\;arms}\times 100$$

The preference in spending time in the novel arm relative to the familiar or start arms is the measure of short-term memory.

#### Spontaneous Alternation

Spatial working memory and exploration were assessed with the Y Maze. ^29–31^ In a maze with multiple arms, mice exhibit the spontaneous behaviour of alternating between arm choices with a greater frequency than re-entering the same arm most recently visited.^31^ The behaviour was recorded (EthoVision XT 9.0, Noldus Information Technology, Wageningen, Netherlands). From the sequence of arm entries, the number of alternation opportunities were counted as the total number of correct and incorrect triads. The percent alternation is the ratio of correct triads to the number of alternation opportunities:

(2) $$Alternation\;(\%)=\frac{Correct\;triads}{Total\;opportunities}\times 100$$

The correct alternation when the mouse entered three different arms consecutively was taken as a measure of spatial working memory.^27^

### Biochemical Studies: lipid extraction and cholesterol quantification

Prefrontal cortices and hippocampi were dissected from brains on ice, rapidly frozen in liquid nitrogen and stored at −80°C. Crude lipids were extracted using a modified Folch method.^32^ Cholesterol content was determined using an enzymatic colorimetric cholesterol assay (Infinity^™^ Cholesterol Liquid Stable Reagent, Thermo Fisher Scientific, Waltham, Massachusetts #TR13421) and normalized to wet tissue weight.

### Immunolabeling of fixed frozen sections

After transcardiac perfusion with PBS and 4% paraformaldehyde, brains were post-fixed for 2hrs and equilibrated in 30% sucrose/PBS. Hemispheres were embedded in OCT and frozen at −80C°. 12 μm coronal cryosections were collected from the rostral cortical areas (+2.2 mm/+1.2 mm from Bregma) and from the hippocampus (−1.2mm/−2.2 mm from Bregma).^33^ Sections adhered to positively charged slides were processed as described. ^25^

### Expansion microscopy of fixed frozen sections by MAGNIFY

MAGNIFY gel monomer solution was prepared as described ^34^. Briefly, cryosections were placed into gelling chambers, after gelation were incubated in softening buffer for 100 min at 95 °C with shaking and washed with 0.1% decaethylene glycol monododecyl ether (C_12_E_10_)/ PBS to remove SDS. After PBS washing the samples were incubated with primary antibodies (Abs) (1:250) in 0.2% Tween 20 in 2X SSC buffer overnight at RT, followed by incubation with secondary antibodies (1:250) for 2 hours at RT.

### Hypoxia Assay

To assess areas of hypoxia, we have used the Hypoxyprobe (2-nitroimidazole hypoxia marker pimonidazole) kit (HP2-100, Hypoxyprobe, Inc., Burlington, Massachusetts) following the manufacturer’s instructions. Mice were injected intraperitoneally with Pimonidazole (60 mg/kg), after four hours anesthetized with 2% isoflurane and perfused intracardially with PBS and 4% PFA. 12 μm cryosections were collected from hippocampus and cortex and stained with an FITC-conjugated Ab which detects conjugates of pimonidazole with protein SH groups in hypoxic cell.

### Single-Cell Transcriptomics

#### Cell suspension preparation and single-cell (sc)RNA-seq library generation

Hippocampi were collected from perfused adult mice, and single-cell suspensions were prepared using the Adult Brain Dissociation Kit (Miltenyi Biotec). Cells were fixed and processed using the Chromium Fixed RNA Profiling workflow (10x Genomics). Four biological replicates per genotype (each consisting of pooled hippocampi from two mice) were analyzed. Libraries were generated using the Chromium Fixed RNA Profiling Reagents Kit for Multiplexed Samples (10x Genomics) and sequenced to a depth which covers more than 10,000 reads per cell.

#### SCT data processing, integration clustering

Raw sequencing data were processed using Cell Ranger multi (v8.0.1, 10x Genomics) and analyzed with Scanpy. Quality control included removal of low-quality cells, cells with elevated mitochondrial transcript content, and predicted doublets identified using scDblFinder. Data integration was performed using scVI. Cell-type annotation was conducted using MapMyCells and refined based on iterative clustering and cluster-specific marker genes identified with Seurat. Ambient RNA contamination was corrected using decontX.

#### Differential Gene Expression and Functional Gene Ontology Enrichment Analysis

Differential gene expression analysis was performed using a pseudobulk approach implemented in muscat, controlling for age and sex. Functional enrichment analysis was conducted using Metascape, and cell-type composition differences were assessed using generalized linear models. Detailed descriptions of sample preparation, quality-control criteria, clustering parameters, cell-type annotation, and statistical analyses are provided in the Supplementary Methods.

### Imaging

Confocal imaging was performed with Nikon A1R and Olympus F4000 microscopes. Brain sections of the four genotypes for each time point were processed together and imaged in sequence using identical laser settings. We imaged the cortical layer (L)1 to L6 in the somatosensory region of the rostral cortex, the medial Prefrontal Cortex (mPFC)^33^ and the CA1 subregion including the alveus, extending medially to the cingulum and laterally 650μm to cover all CA1 layers.^33^ We captured one Field of View (FOV) per section imaged at 20X with Z stacks of 10-25 steps with 1 μ step for cellular and membrane staining. Synaptophysin and TREM2 staining was imaged with 40X objective with 1.5 software zoom with for Olympus and 60X objective for Nikon with Z stacks for 10-25 steps with a 1μ step respectively.

Imaging of MAGNIFY post-expansion sections was performed using a Nikon Eclipse Ti2 epifluorescence microscope equipped with a CSU-W1 spinning disk confocal module and an Andor 4.2 Zyla sCMOS camera and controlled by NIS-Elements AR 5.21.03 64-bit software. Images were taken with CFI Plan Apo Lambda 4× (0.2 NA), CFI Apo LWD Lambda S 20×WI (0.95 NA), and CFI Plan Apo VC 60×C (1.2 NA) water immersion objective lenses. We imaged the CA1 subregion and the somatosensory cortex as described above using 20X and 60X objectives. We captured 3 FOVs per section (*n*=two/mouse sample) with the 60X objective.

### Imaging Data Analysis

The Maximum Intensity Projection (MaxIP, NIS Element, Nikon) or Projection Z (cellSENS FV, Evident) was created from the individual Z stack images. 2D image quantification was performed in Fiji using integrated density for myelin membrane markers and for GFAP cellular profiles and manually counting positive cells in cortex or CA1 subregion. Quantification of myelin protein markers, MBP and MOG was performed in a preset region of interest (ROI) by the IntDen feature in Fiji. The CA1 ROI was 400 μm length and 250μm in width across the entire CA1 subregion including alveus and all CA1 layers. The sensory cortex ROI was 300 μm length and 250μm of width across L2-3 and L4-6.

The plaque burden was quantified by: number as the counts of 6E10 stained accumulations >100 μm^2^, the area occupied by plaques (%) and the integrated density by using 3D Object Counter v2.0.1 plug-in in Fiji. TREM2+ and synaptophysin puncta were quantified with Imaris (v10.0.1, Oxford Instruments, Abingdon, UK) using the surface and spot functions. DAPI, Iba1 and 6E10 were rendered by Surface and TREM2 as spots with 4 μm diameter. TREM2+ /Iba1+cells were identified by filtering with the distance of the spot to the nucleus set at 3 μm. 6E10 accumulations <1000 μm^3^ in volume were excluded during surface creation. The TREM2+Non-Plaque Associated Microglia was obtained by subtracting the TREM2+ Plaque Associated Microglia defined by direct contact to 6E10 staining from the total TREM2+ microglia. TREM2+/Iba1+ cells at the plaque niches defined as a circle of 50 μm diameter around the 6E10 staining were manually counted in three niches. In both pre-expansion and post-expansion images synaptophysin was rendered as spots with 2 μm diameter. The number of synaptophysin spots was measured at the plaque niche and in a comparable size area free of 6E10 staining. The ratio of synaptophysin spots at plaque to those in the plaque free area represented the percent loss.

DAPI and NeuN were rendered by surface and 4-HNE structures as spots with 2 μm diameter. 4-HNE+/NeuN+ cells were identified by filtering with the distance of the spot to the nucleus set at 1 μm. For hypoxic neurons, we measured the fluorescence per area by integrated density with Fiji and counted fluorescently labelled cells identified by DAPI in the CA1 subregion and normalized by the volume.

### Statistics and Reproducibility

For behavioural testing, the experimenter was blinded to the identity of the subjects. Supplementary Table 1 shows the number of mice for each assay and the source of attrition. The ratio of M:F was 60:40 at 6 months, 50:50 at 12 months and 40:60 at 16 months. Whenever possible for quantitative imaging, the experimenters were blinded to the identity of the samples. For all imaging datasets, we analysed two to three sections per mouse over a set of four mice per genotype. For expansion microscopy, we analysed two sections per mouse and imaged 3 FOVs/per section. Each technical replicate was analysed separately and averaged to obtain the mean per biological replicate. The biological replicates were then pooled for final genotype analysis. Sample sizes and number of mice were based on means and SEM generated in our laboratory and our published work.^24,35,36^ Groupwise normality assessed using Shapiro-Wilk test showed deviation from normality in some of the assays. Unequal variances were detected using the Brown-Forsythe test across groups in several assays. To best fit the statistical analyses to the structure of the data, we have performed Welch’s ANOVA. For pairwise post-hoc group comparisons, Welch two-sample t-tests were computed, and *P*-values were adjusted for multiple comparisons using Holm’s procedure within each assay × age family of pairwise comparisons. For two group comparisons, we have used Welch’s t test. The data were plotted in GraphPad Prism Version 10.4.1.

## Results

### Spatial working memory is preserved in the aged AD/cKO and hippocampal plaque burden is reduced

We crossed the *App*^NL-G-F^ (AD), which carries three human mutations in the mouse App gene under the endogenous App promoter and is not subject to the overexpression seen in other mouse lines,^26^ with the *Fus*^fl/fl^/*Cnp*^cre/+^(cKO), a developmentally and physiologically regulated hypermyelinating mouse model to generate the AD/cKO line.^24^ We reasoned that the AD/cKO line would provide a more physiological context in which to study the contribution of myelin and OL to AD disease progression.

We conducted all studies in the AD, AD/cKO, cKO and WT (Fig. 1A), with WT and cKO serving as age matched controls and the cKO as genotype specific control for the AD/cKO. Immunohistochemistry and biochemical studies were performed in the hippocampus, critical component of the integrated memory circuit^37–39^ and somatosensory (S1) cortex, which is involved in higher cortical functions ^33^ at time points selected on the basis of previous pathology characterization (Fig. 1A).^26,40^

Mice 6, 12 and 16 months old *(n*=7-17, Supplementary Table 1) were tested for short-term spatial memory with the Novel Spatial Recognition and spatial working memory with the spontaneous alternation in the Y maze. ^27^ Since different ages of symptom onset were previously reported,^41–44^ we first tested the 16 months old subjects and, once deficits were identified, we examined 6- and 12-months old mice. The same cohort of mice was tested at 6- and 12-months of age (Supplementary Table 1). In the novel spatial recognition test, the 16 months old AD and AD/cKO mice spent similar time exploring the novel arm as the WT and cKO at all ages (Fig. 1B), showing no decline in short term memory. In the spontaneous alternation test (Fig. 1C), the 16 months old AD mice showed a statistically significant reduction in spontaneous alternation relative to the age matched AD/cKO (Fig. 1C, *P*=0.0005) and a reduction non-statistically significant relative to age matched WT (Fig. 1C, *P*=0.06). The AD/cKO performed at the same level as the WT (Fig. 1C, *P*=0.927) and cKO (Fig. 1C, *P*=0.827), indicating that spatial working memory is preserved in the AD/cKO vs AD. WT and cKO performed similarly. No differences were detected at 6 and 12 months of age (Fig. 1C). The symptom onset in our line was later than that reported previously and is likely explained by the effect of background genes and subtle differences between mouse sub-strains.^45^ A sex effect on behaviour was previously reported.^44^ While we included males and females, the numbers were not balanced and powered to reliably evaluate sex as a variable. The sexes are graphically shown in Fig. 1 and Supplementary Fig. 1.

Hyperactivity and reduced anxiety behaviour were reported in the *App*^NL-G-F^.^43,46^ We tested hyperactivity by counting the number of entries in the arms of the Y maze and the total distance travelled in the Elevated Plus Maze (EPM), and anxiety by measuring the time spent in the open vs closed arm in the EPM. Hyperactivity and reduced anxiety were present in 16 months old AD and AD/cKO and no difference was detected (Supplementary Fig. 1).

We analysed age x genotype interaction with a Linear Mixed Effect (LME) model for the behavioural data at 6 and 12 months. The LME did not show genotype x age interactions for any of the measures (Supplementary Fig. 2). An age effect was present for all genotypes, as illustrated by the slopes (Supplementary Fig. 2A) and was more prominent for the WT in measures of hyperactivity. A main effect of age was significant within genotype for the hyperactivity measures and a genotype effect for the spontaneous alternation at 16 months (Supplementary Fig. 2B). No differences were detected in either hyperactivity and anxiety between the WT and the cKO, as previously published. ^24^

Next, we examined whether preserved working memory was associated with reduced Aβ plaque burden, measured by number of plaques per field of view (FOV), area occupied by plaques and the integrated density in the hippocampus and cortex (Fig. 1D and E). Plaque burden, which increased with disease progression in CA1 and cortex, was statistically lower in the CA1 of the AD/cKO vs AD at 16 months of age (Fig. 1 D and E, counts *P*=0.019; area: *P*=0.011; IntDen: *P*=0.029), while it was not statistically different in the AD/cKO vs AD S1 cortex (Fig. 1 D and E) and medial prefrontal cortex ^47^ (Supplementary Fig. 4A). The data show that preserved spatial working memory at 16 months of age in the AD/cKO is associated with decreased plaque burden in the CA1, but not in cortical regions relevant to cognition and memory.

### Differential regulation of microglia density and protective state in the AD/cKO CA1 and cortex

Microglia activation is an early event in AD pathology^48^ and microglia states change in response to the environment and affect disease progression. ^49–52^ We have examined the temporal course of microglia cell accumulation by counting the number of Iba1+ cells in the cortex and CA1. In both AD and AD/cKO microglia density increased in cortex and CA1 as the mice aged (Fig. 2 B, D). Iba1+ cell density was significantly higher in the CA1 of the AD vs AD/cKO mice at 12 (Fig. 2A, B, *P*=0.0051) and 16 months of age (Fig. 2 A, B, *P*=0.0007). In contrast, no statistically significant differences were detected in cortex of the AD vs AD/cKO at 12 and 16 months (Fig. 2 C, D) and the mPFC at 16 months old (Supplementary Fig. 4B).

TREM2+ microglia have strong phagocytic capacity, cluster at the plaques where they reduce Aβ accumulations,^51^ and present an anti-inflammatory and protective state.^53^ We measured TREM2+/Iba1+ microglia density at the plaque niches in the AD and AD/cKO CA1 and cortex. TREM2+/Iba1+ cells were statistically higher at the niche of the AD/cKO CA1 vs AD at 12 (Fig. 2 E, F, *P*=0.038) and 16 months of age (Fig. 2 E, F, *P*=0.0003), while they were increased only at 16 months in the cortex (Fig. 2 E, F, *P*=0.0008) and mPFC (Supplementary Fig. 4C *P*=0.0061). No statistically significant difference was present in 6 months AD/cKO vs AD in cortex (Fig. 2E, F) and CA1 (Fig. 2E, F). The total number of TREM2+, plaque associated, defined as TREM2+ microglia associated with Aβ deposition and non-plaque associated TREM2+ were not statistically different in 6-, 12- and 16-months old AD and AD/cKO CA1 (Supplementary Fig. 3A) and cortex (Supplementary Fig. 3B). The data indicates that the higher number of TREM2+ microglia at the plaque niche of the AD/cKO is not accounted for by an increase in total TREM2+ microglia or a shift from the non-plaque associated to plaque associated microglia and suggests that local signalling at the plaque niche stabilizes TREM2+ microglia state.^54^

While TREM2+ microglia may lead to preservation of memory by reducing plaque burden, ^51^ plaques were reduced at 16 months only in CA1 not in cortex despite the high number TREM2+ microglia at the niches in both. We postulated that TREM2 microglia exert a protective effect which is independent from plaque burden. We evaluated synaptic integrity by quantifying density of pre-synaptic structures in the AD/cKO vs AD by synaptophysin staining, previously found to be diminished at the plaques of the AD mouse.^26^ Standard immunofluorescence staining revealed a statistically significant retention of synaptophysin in 16 months AD/cKO vs AD CA1, cortex (Supplementary Fig. 5) and mPFC (Supplementary Fig. 4C). To enhance detectability and resolution of the synaptophysin puncta, we have measured synaptophysin by expansion microscopy with MAGNIFY which resulted in 3.2X amplification [Fig. 2G, H, Supplementary Fig. 5 and Imaris 3D movie Supplementary Video 1 AD (available at https://doi.org/10.6084/m9.figshare.32671602) and Supplementary Video 2 AD/cKO (available at https://doi.org/10.6084/m9.figshare.32682372)]. Synaptophysin loss was significantly reduced in the 16 months old AD/cKO CA1 and cortex vs AD (Fig. 2G, H, 50% reduction, *P*=0.03), while no difference was present at 12 months (Fig. 2G, H), confirming the pre-expansion data (Supplementary Fig. 5A).

Together, the data indicate that TREM2^+^ microglia at the plaque niches of the aged AD/cKO are associated with retention of presynaptic structures in both cortex and CA1 independently from amyloid plaque burden.

### Astrocytic activation increases with disease progression in cortex and CA1 of the AD and AD/cKO

Astrocytes play a critical role in the maintenance of brain homeostasis. ^55–57^ and undergo several morphological and gene expression changes in AD progression.^58^ We measured markers of astrocytic activation and found that they increased similarly in cortex and CA1 of the AD and AD/cKO except for the GFAP+ profiles that were higher in the cortex of the AD/cKO vs AD (Supplementary Fig. 6C, D). The data do not suggest a genotype effect on astrocyte activation.

### Myelin density and cholesterol content are differentially regulated in the AD/cKO CA1 and cortex

Myelin thickness and cholesterol content are elevated in cKO white matter tracts. ^24^ Here, we have measured compact myelin density by staining for MBP and PLP,^59^ and new myelin segment deposition by staining for MOG, which resides in the outermost layer of myelin, in CA1 and cortex. ^60,61^ In the CA1, there was a physiological increase in MBP and MOG expression from 2 to 6 months in WT and cKO with stable levels at 12 and 16 months of age (Fig. 3A, B, C). MBP expression was higher in the cKO and AD/cKO vs WT and AD, consistent with *Fus* depletion driven increase in myelin thickness, the increase was statistically significant only in the cKO vs WT at 6 (Fig.3 A, B, C, *P*=0.045) and 12 months (Fig. 3A B C, *P*=0.042). MOG was not increased indicating absence of new myelin segment formation (Fig. 3 A, B, C).

In the L2-3 cortex, where a large proportion of axons are sparsely myelinated and adaptive myelination is easily detected in the adult brain,^62^ MBP and MOG densities increased from 2 to 6 months in WT, cKO, AD and AD/cKO (Fig. 4 A,B,C). MBP and MOG densities were statistically higher in the cKO relative to WT (Fig. 4 A,B,C, MBP *P*=0.027; MOG, *P*= 0.015) and in the AD/cKO relative to AD (Fig. 4 A,B,C, MBP, *P*=0.005 and MOG, *P*=0.015). MOG gradually declined from 6 to 16 months, while MBP sharply declined at 12 months (Fig. 4 A,B,C). In L4-6, myelination is high, MBP and MOG densities were similar in all genotypes (Supplementary Fig. 7A). The data suggests that *Fus* depletion in OL leads to increased myelin thickness and new myelin segment deposition in L2-3.^78^ MBP and MOG staining was not reduced in the cortex and CA1 of the AD relative to the WT and AD/cKO relative to cKO (Fig. 3 and Fig.4).

The density of the major intrinsic myelin protein, PLP, in cortex L2-3 was not statistically different in the AD, AD/cKO vs WT and cKO (Supplementary Fig. 7B). Western blot analysis of PLP and MBP in hippocampus and cortex lysates of WT, cKO, AD and AD/cKO did not reveal any differences in protein levels (Supplementary Fig. 7C, D). The increase in MBP by immunostaining performed in L2-3 reflects adaptive myelination and may not be detectable by WB of the whole cortex lysate.

Taken together, the studies did not reveal significant myelin loss in the AD vs WT, in keeping with previously published data,^22^ and in the AD/cKO vs cKO. An increase in compact myelin measured by MBP immunostaining was present in CA1 and in the L2-3 of the AD/cKO and cKO cortex.

Cholesterol content is higher in the cKO white matter tracts relative to WT mice. ^24^ We have measured cholesterol and cholesterol esters with a colorimetric assay in lipids extracted from hippocampus and cortex of WT, cKO, AD and AD/cKO. Cholesterol was significantly higher at 2 (Fig. 5A, *P*=0.034) and 6 months (Fig. 5A, *P*=0.0003) in the AD/cKO vs AD hippocampus but not at 12 (Fig. 5A, *P*=0.404) and 16 months (Fig. 5A, *P*=0.656). Cholesterol trended up in the hippocampus of the cKO vs WT at 6 (Fig. 5, *P*=0.061) and 16 months (Fig. 5A, *P*=0.061) but not at 2 (Fig. 5, *P*=0.094) and 12 month (Fig. 5A, *P*=0.111). No differences were present in the cortex of the four genotypes, except for cKO vs WT at 16 months (Fig. 5B, *P*=0.03). The spatial differences in cholesterol content between CA1 and cortex may be explained by the heterogeneity of mature OL in hippocampus vs cortex ^63^ and regional restriction in cholesterol metabolism. ^64^

### Transcriptional profiles in AD/cKO hippocampus reveal significant gene changes in MOL and microglia

To elucidate how *Fus*-deficient OL mediate such widespread effects on microglial cellular state, presynaptic structure loss at the plaque niche, and progression of AD pathology in the hippocampus, we performed a single-cell transcriptomic (SCT) analysis on hippocampal cell suspensions isolated from WT, cKO, AD, and AD/cKO mice at 4–5 months of age (Fig. 6A).

Following quality control and subsequent clustering and annotation using reference datasets from the Allen Mouse Brain Atlas, we retained 115555 high-quality cells which were hierarchically classified into 26 major cell types that were consistently represented across samples (Fig. 6B, Supplementary Fig.8A, B). We observed a marked depletion of *Fus* expression in MOL and a moderate reduction in *Fus* in committed oligodendrocyte precursors (COP), newly formed oligodendrocytes (NFOL), and myelin-forming oligodendrocytes (MFOL), in both cKO and AD/cKO compared to WT and AD mice consistent with the CNP-cre promoter expression (Fig. 6C).

MOL and microglia exhibited the largest numbers of Differentially Expressed Genes (DEG) with MOL most affected in the *Fus* knockout contrasts (AD/cKO vs WT, cKO vs WT, AD/cKO vs AD), whereas microglial changes were evident in AD and AD/cKO comparisons versus WT (Fig. 6D). We identified 432 upregulated and 433 downregulated genes in AD/cKO vs AD MOL. In the cKO vs WT comparison, 134 genes were upregulated and 111 downregulated in MOL. Only 47 genes were upregulated and 48 downregulated in AD/cKO MOL relative to WT (Fig. 6D). These findings indicate a strong impact of *Fus* depletion specifically in the AD background, suggesting a genotype-to-genotype interaction. Gene Ontology (GO) enrichment analysis of the DEG in AD/cKO vs AD MOL revealed significant upregulation of genes involved in metal ion response, amyloid precursor protein catabolic processes, oxidative phosphorylation, aerobic respiration, respiratory electron transport, redox homeostasis, and detoxification pathways (Fig. 6 E, F). Genes associated with cholesterol metabolism and lipid biosynthesis were significantly downregulated (Fig. 6 E, F).

In microglia, we identified 541 upregulated and 196 downregulated genes in AD mice relative to WT, and 561 upregulated and 263 downregulated genes in AD/cKO mice relative to WT (Fig. 6D). GO enrichment analysis showed that most upregulated genes in AD vs WT and AD/cKO vs WT comparisons were associated with inflammatory responses and cellular activation pathways, indicating a robust App-dependent transcriptional program (Supplementary Fig. 8C, D). Downregulated genes were enriched for pathways related to the regulation of protein phosphorylation.

Most of the up and downregulated genes in MOL are unique to the AD/cKO vs cKO MOL. Remarkably, oxidative phosphorylation genes of mitochondrial complex 1 (Ndufa1, 3 and Ndufb5, mt-Nd5), antioxidant (SOD1, Gstp1, Gss), electron transport chain are exclusively upregulated in the AD/cKO vs AD but not in the cKO vs WT (Fig. 6F). Some of the genes involved in OL differentiation (Ncstn and Anxa5) are upregulated in both AD/cKO and cKO MOL (Fig. 6F), whereas others (Myef2, Cdk5 and Psenen) are exclusively up regulated in AD/cKO MOL, suggesting a stronger maintenance of OL identity and differentiation vs the AD MOL. Ncstn and Psen2 regulate Notch processing important for OL differentiation and are involved in App processing, suggesting a potential cross regulation of App and other membrane protein processing.^65,66^ Gelsolin which participates in myelin wrapping,^67^ and Cln3 involved in membrane, lysosome trafficking and cytoskeleton organization are up regulated in AD/cKO vs AD and vs WT and cKO vs WT (Fig. 6F).

Genes involved in fatty acid (FA) metabolism, fatty acid chain elongation and desaturation and sterol export, are downregulated in both AD/cKO and cKO MOL. Some of these genes fall into the GO enrichment category of cholesterol metabolism by affecting FA synthesis, however, the main cholesterol synthesis enzymes, namely Hmgcr and Hmgcs are not downregulated (Fig. 6F).

To determine gene expression changes reflected altered microglial and MOL subtype proportions, we resolved the dataset into 37 high-resolution cell types, capturing seven MOL subclusters (MOL_1 to MOL_7) and six microglial subclusters (Microglia_1 to Microglia_6) (Supplementary Fig. 9A, B). Microglia_1 through Microglia_3 expressed high levels of homeostatic markers (*P2ry12* and *P2ry13)*, whereas Microglia_4 and Microglia_5 exhibited reduced expression of homeostatic markers and increased expression of activation-associated genes (*Lpl* and *Itgax)*, indicative of activated states. Microglia_6 displayed a proliferative signature, marked by elevated *Mki67* expression. Clusters Microglia_4 and Microglia_5 were enriched in both AD and AD/cKO groups (Supplementary Fig. 9C), identifying them as disease-associated microglial states (Supplementary Fig. 9C).

Proportional analyses revealed a significant increase in Microglia_4 and Microglia_5 in both AD and AD/cKO relative to WT, accompanied by a significant reduction in the homeostatic Microglia_1 population (Supplementary Fig. 9D), with no significant differences between AD and AD/cKO (Supplementary Fig. 9D). The seven MOL clusters exhibited distinct transcriptional signatures (Supplementary Fig. 9C). MOL_3 showed elevated expression of genes involved in oligodendrocyte differentiation (*Enpp6*)^68^ and cytoskeletal organization (*Anln*). MOL_7 was characterized by high expression of *Cntn1* (contactin), which is essential for myelin–axon interactions, as well as *Man1a* and *Pcdh7*, markers associated with late-stage, remyelinating mature oligodendrocytes (bioRxiv, Dolan et al., 2025). MOL_6, defined by expression of *Apoe* and metallothionein genes (*Mt2*, *Mt3*), was significantly increased in cKO mice relative to WT (Supplementary Fig. 9D). MOL cluster proportions did not differ significantly between AD and AD/cKO groups (Supplementary Fig. 9B). Taken together, the SCT data have revealed a major presence of DEG in energy metabolism, antioxidant and OL differentiation in AD/cKO vs AD and have uncovered a potential role of these genes in reducing neuronal damage.

### Oxidative damage is reduced in the AD/cKO compared to AD

We have investigated whether enhanced energy metabolism genes result in improved neuronal health. We have measured hypoxic damage caused by Aβ-oligomer driven capillary vasoconstriction in the AD/cKO by quantifying pimonidazole conjugates with SH protein groups in neurons. ^69,70^ The number of hypoxic neurons was increased in both AD and AD/cKO vs WT and cKO at 4 months of age (Fig. 7A). The count of hypoxic neurons was trending down in CA1 (Fig. 7A, *P*=0.202) and cortex (Fig. 7A, *P*=0.220) of the AD/cKO vs AD, but did not reach statistical significance.

To assess reactive oxygen species (ROS) damage, we measured lipid peroxidation by quantifying 4-Hydroxynonenal (HNE)+ adducts ^71–73^ in 6, 12, 16 months old WT, cKO, AD and AD/cKO. A significant increase in 4-HNE+ cells was detected at 12 and 16 months CA1 (Fig. 7C) and cortex (Fig. 7E) in the AD vs WT and AD/cKO vs cKO. The 4-HNE+ cells were statistically lower in the 16 months old AD/cKO vs AD CA1 (Fig. 7 B, C, *P*=0.0179) and cortex (Fig. 7 D,E, *P*=0.0022). The data indicate reduced overall cellular oxidative damage in the AD/cKO.

Next, we assessed neuron oxidative damage. An increase in 4-HNE+/NeuN+ cells was present in CA1 (Fig. 7G) and cortex at 12 and 16 months (Fig. 7I). However, HNE+/NeuN+ cells were significantly lower in the 16 months old AD/cKO CA1 (Fig. 7F, G *P*=0.0047), cortex (Fig. 7H, I *P* =0.0036) and mPFC (Supplementary Fig. 4F, *P*=0.0171) vs AD. Taken together, the data indicate reduced oxidative damage in neurons of the AD/cKO.

## Discussion

Disruption of oligodendrocyte and myelin support to neurons is an important contributor to AD pathobiology and represent potential targets for disease modification strategies. The current studies have uncovered enhanced energy metabolism gene expression in AD/cKO OL associated with preservation of spatial working memory. The upregulation of energy metabolism and antioxidant genes present in AD/cKO OL occurs early in disease prior to plaque accumulation, consistent with an adaptive response aimed at enhancing OL energy supply, antioxidant defence and maintenance of trophic support to neurons.

Clustering of TREM2+ microglia at the aged plaque niches^74^ was increased in the AD/cKO and was associated with preservation of presynaptic structures in cortex and hippocampus. TREM2+ microglia acts as a barrier to protofibrillar Aβ deposition,^51,75 60,61,95^ reduces Aβ induced dystrophic neurites, and secretes anti-inflammatory cytokines.^76,77^ Restoration of axonal transport in rescued neurites^78^ and secretion of anti-inflammatory cytokines by TREM2+ microglia^54^ may enhance presynaptic integrity at the plaque niches of the AD/cKO. Antibody mediated stabilization of TREM2 increased TREM2+ microglia at the plaque niches^79^ and improved performance in cognitive testing of treated AD mice.^80^ These findings have led to testing the clinical application using a TREM2 targeting monoclonal antibody agonist in a Phase II trial with early AD patients. While the trial failed to reach primary endpoint,^81^ it highlighted the need to better evaluate and modulate the state of TREM2 microglia.^82^

While TREM2 microglia limit amyloid deposition,^51^ a reduction of plaque burden is present only in the AD/cKO hippocampus but not in cortex and mPFC, uncoupling plaque burden from synaptic structure preservation. Earlier TREM2+ microglia clustering in the hippocampal plaques and different rate of Aβ production and deposition by OL^83^ may account for lower plaque burden. The uncoupling of plaque burden and synaptic preservation suggests a role of plaque-independent mechanisms such as the toxic effects of soluble Aβ oligomer^84^ and the early presence of OL oxidative damage in AD brains,^85,86^ reduced by *Fus* depletion.

The higher TREM2+ microglia at the plaque niche is not due to a shift in non-plaque associated to plaque associated TREM2+ microglia,^87^ and suggests local signals at the plaque niche that maintain the activated state and prevent microglia senescence.^88^ A major signalling ligand-receptor pair is the Csf1-Csf1r acting through autocrine signalling in microglia ^54^ as well as a paracrine signalling from astrocytes and OL. ^89,90^ Since OL are a major source of Csf1 in adult brain and development,^89^ it is possible that *Fus* depleted MOL at the plaque niche secrete Csf1 and promote TREM2+ microglia.^88^ Future spatial transcriptomics will elucidate OL-microglia communications at the plaque niche.

Genes involved in OL differentiation and membrane transport are upregulated in *Fus* depleted AD OL ensuring maintenance of the OL differentiation state and the transport of myelin proteins to the myelin membranes. A *Fus* dependent increase in compact myelin by MBP staining was present in AD/cKO hippocampus and cortex, while PLP was not increased. The discrepancy may reflect the high abundance and stability of PLP limiting detection of changes by immunohistochemistry. Our studies did not reveal a statistically significant loss of compact myelin in the AD hippocampus and cortex up to 16 months. This limits the interpretation of the effect of greater myelin deposition in the AD/cKO. Previous studies using electron microscopy have shown either loss,^91^ or preservation of myelin^21^ in this AD model. It is possible that discrepancies between studies reflect subtle background gene variations ^45^ and the presence of more nuanced defects of myelin axon interactions rather than loss of compact myelin.^23^

The increase of neuronal oxidative damage is likely to result from neuron intrinsic energy demands, antioxidant failure and loss of extrinsic support from glial cells.^92^ How *Fus* depleted OL ameliorate neuronal oxidative damage in the aged AD/cKO remains to be elucidated. OL rescue axonal mitochondria by transcellular delivery of Sirtuin 2 a NADH dependent deacetylase. ^93^ This transfer is likely to fail in stressed OL and may be preserved in *Fus* depleted OL leading to reduced neuronal oxidative damage. Hypoxia caused by pericyte mediated capillary constriction is an early event in AD, which leads to hypoxic damage to neurons.^69,70^ Mature oligodendrocytes associate directly with blood vessels and are an integral component of the blood vessel wall.^94,95^A downward trend in the number of hypoxic neurons in the AD/cKO hippocampus and cortex, although not statistically significant, suggests a potential beneficial effect of AD/cKO OL in reducing hypoxia.

Lipids are critical for myelin maintenance and neuron and synapse health.^96^ We found that cholesterol content is higher at early stages in the hippocampus of the AD/cKO vs AD, while it did not reach statistical significance in the cKO vs WT comparisons, suggesting genotype-to-genotype interaction in the AD/cKO or the contribution of astrocytic cholesterol.^97^ The decline in cholesterol in the AD/cKO OL at late stages of AD may indicate reduced metabolic capacity in aged *Fus* depleted AD OL. Unlike hippocampus, the cholesterol was not increased in the cortex of cKO and AD/cKO. This spatial difference may reflect regionally regulated myelin cholesterol and lipid composition.^64^ CA1 hippocampus is enriched in OL with high expression of lipid biosynthetic enzymes, while S1 cortex is enriched in OL that express synapse components and cytoskeletal genes.^63^ OL lipid metabolism correlates with axon size^64^ and is regulated by factors such as neuron signal, myelin demand from axon and neural circuit activity.^23^

In summary, we show that upregulation of energy metabolism and antioxidant gene expression and higher myelin density induced by *Fus* depletion in the context of App gene mutations are associated with preserved memory, reduced neuronal oxidative damage and a more protective cellular environment by affecting microglia state at the plaque niche. While *Fus* has not been linked to AD in genome wide studies, leveraging a *Fus* cKO in OL has advanced our knowledge of OL mediated protective role in AD models. Pathways such as energy metabolism, antioxidant, endosomal protein transport and lipid metabolism have emerged as important targets for interventions. We propose that OL represent an important “hub” that mediates homeostatic support through multiple mechanisms and cellular targets and offer new potential AD disease modifying strategies.

## Supplementary Material

supplementary zip

Supplementary material is available at *Brain* online.

## Figures and Tables

**Figure 1:**
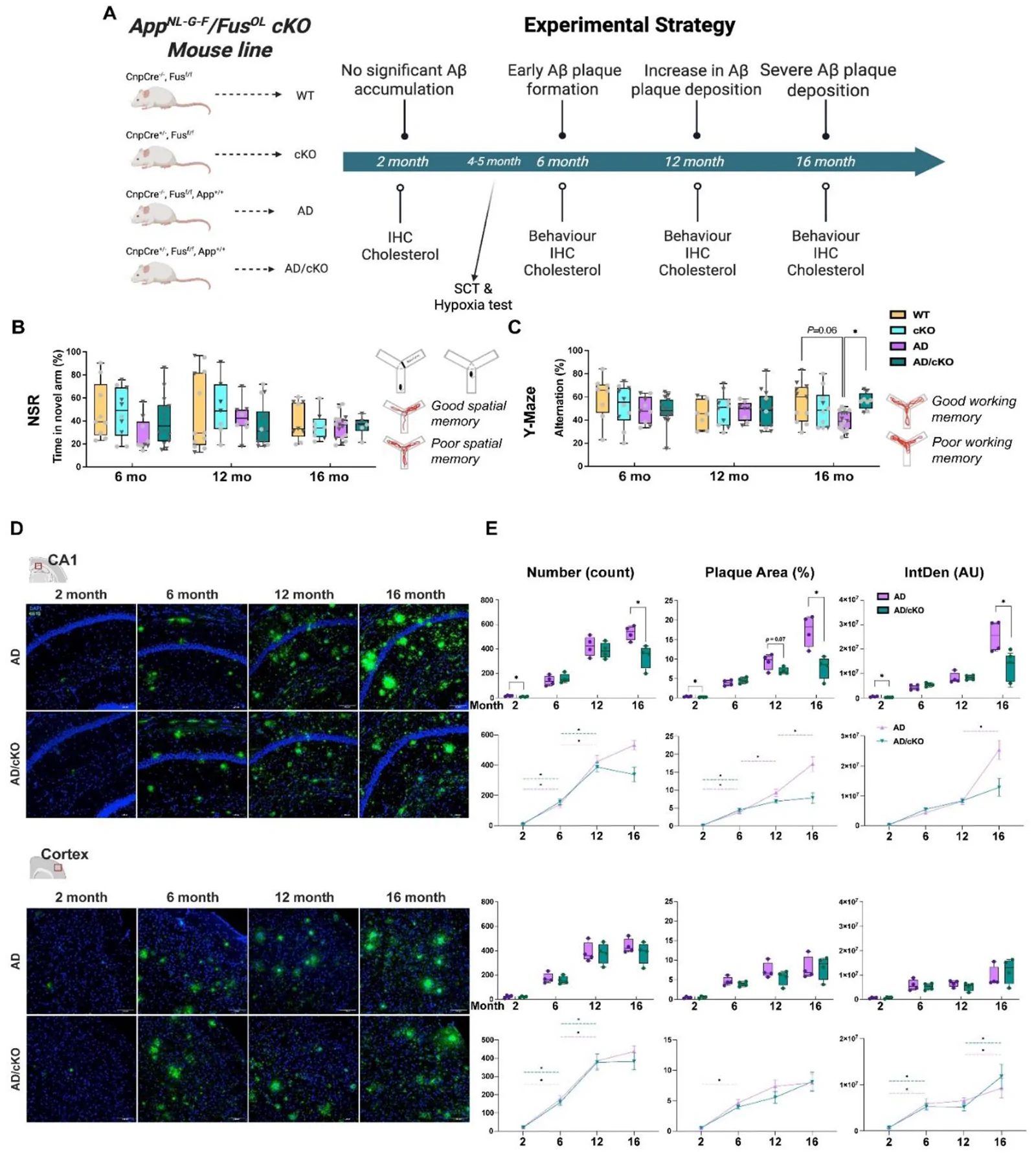
Aged AD/cKO mice preserve spatial working memory and show reduced plaque accumulation in the CA1 subregion of the hippocampus. **A.** Graphic representation of the mouse line indicates the four genotypes, WT, cKO, AD and AD/cKO and the Experimental Strategy shows the timeline and the experimental approaches. IHC = immunohistochemistry; SCT = single-cell transcriptomics. **B.** The graphs show the time (%) that each mouse spent in the novel arm after a trial period in which one of the arms was closed (Novel Spatial Recognition). The circles represent females and the triangles males. There are no statistically significant differences between genotypes. The graphic representation shows the Y maze with the arm blocked used in trial 1 and the Y maze with the new arm open used in trial 2 and the read outs. **C**. The graphs show the spontaneous alternation (%) in the Y maze for each mouse. The AD mice show a reduction in percent alternation vs WT that did not reach statistical significance (39.47%±2.07 vs 55.51%±5.16, *P*=0.063). The percent alternation in AD/cKO mice shows no difference vs WT and cKO (56.65%±2.54 vs 55.51%±5.16, *P*=0.927; 56.65%±2.54 vs 50.03%±5.20, *P*=0.828) and are significantly higher vs the AD mice (56.65%±2.54 vs 39.47%±2.07, *P*=0.0004). The graphic representation shows the Y maze and the read outs. **D**. Representative 20X confocal images of cryosections stained with 6E10 to label β amyloid and DAPI to identify nuclei in the CA1 subregion and the sensory cortex of 2-, 6-, 12- and 16-months old AD and AD/cKO. **E**. The graphs show the plaque counts, the area occupied by the plaques and the integrated density (intensity of fluorescence per area) per FOV. All three readouts are significantly lower in the CA1 of 16 months old AD/cKO vs AD (counts: 338.2±48.4 vs 532.0±29.9, *P*=0.019; area: 7.87%±1.48 vs 17.33%±2.04, *P*=0.011; IntDen: 1.28x10^7^±3.01x10^6^ vs 2.53x10^7^±3.19x10^6^, *P*=0.029). No differences were detected at 6 and 12 months. No significant differences were detected in any of the readouts in the cortex of AD/cKO and AD at 6, 12 and 16 months. The rate of plaque accumulation was similar in the AD and AD/cKO up to 12 months in the CA1 and up to 16 months in the cortex. mo = month. Figure partially created in BioRender. Tung, T. (2026) https://BioRender.com/fbxm4xy.

**Figure 2:**
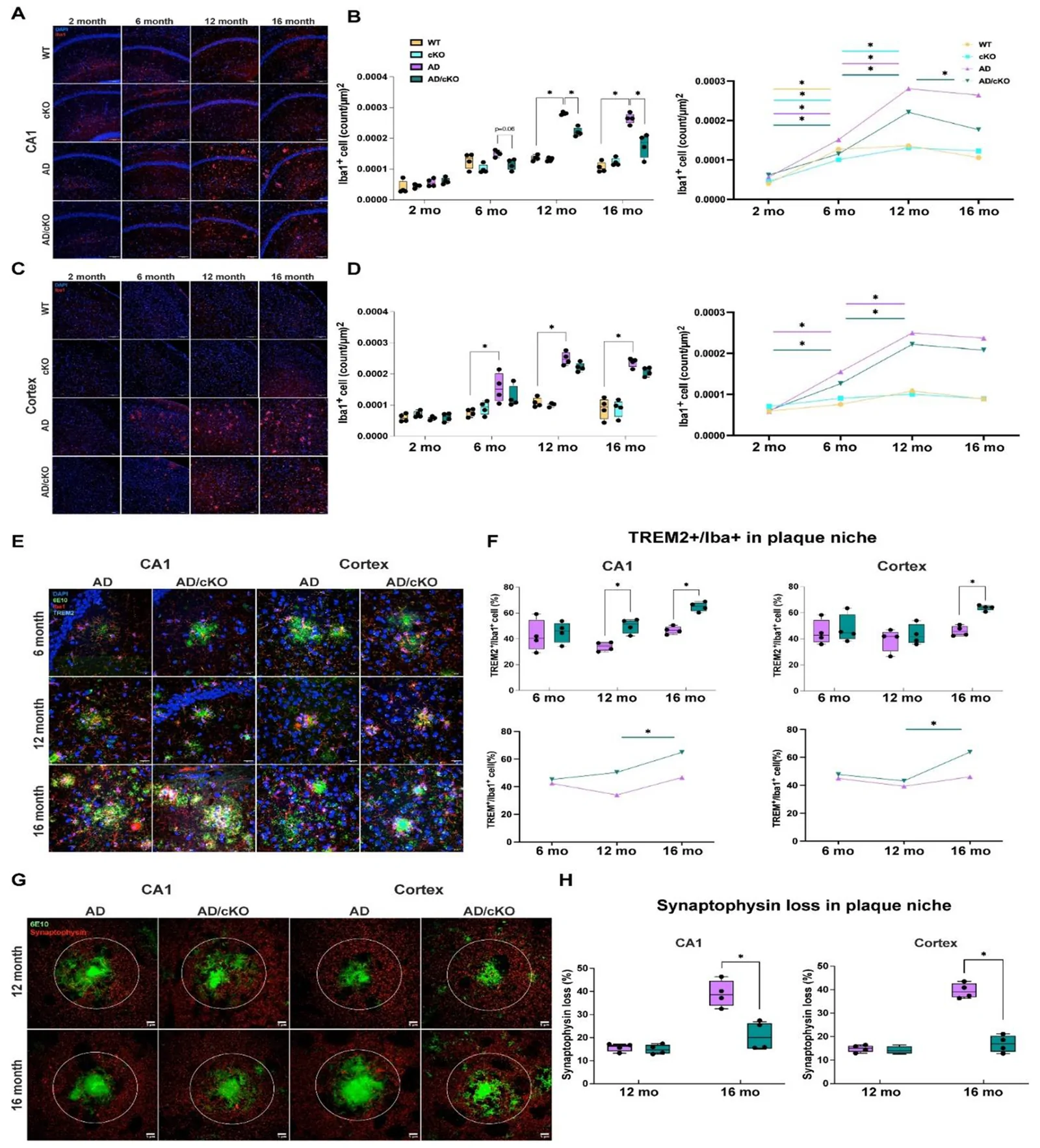
Analysis of microglia density, TREM2+ microglia at the plaque niche and pre-synaptic structures in CA1 and cortex. **A.** Representative 20X confocal images of Iba1 stained cells in the CA1 of 2-, 6-, 12- and 16-months old WT, cKO, AD and AD/cKO. **B.** The graphs show the Iba1+ cell counts in the FOV of 3 sections in CA1 of 2-, 6-, 12- and 16-months old WT, cKO, AD and AD/cKO (*n*=four), * *P*<0.05. Line plot shows the temporal course of Iba1+ count increase in CA1 of 2-, 6-, 12- and 16-months old WT, cKO, AD and AD/cKO. **C.** Representative 20X confocal images of Iba1 stained cells in cortex of 2-, 6-, 12- and 16-months old WT, cKO, AD and AD/cKO. **D.** The graphs show the Iba1+ cell counts in the FOV of 3 sections in cortex of 2-, 6-, 12- and 16-months old WT, cKO, AD and AD/cKO (*n*=four), *P<0.05. Line plot shows the Iba1+ count increase in the cortex of 2-, 6-, 12- and 16-months old WT, cKO, AD and AD/cKO. **E.** Representative 40X confocal images of 6E10 (green), Iba1 (red) and TREM2 (cyan) stained CA1 and cortex of 6-, 12- and 16-months old AD and AD/cKO. White arrowheads point to TREM2+/Iba1+ cells. **F.** The graphs show the TREM2+/Iba1+ cells (%) in 3 plaque niches from three sections in CA1 and cortex (*n*=four), * *P*<0.05. Line plot shows the temporal course of TREM2+/Iba1+ percent increase at plaque niches of the CA1 and cortex of 6-, 12- and 16-months old AD and AD/cKO. **G.** Representative post-expansion images captured by epifluorescence microscopy with 60X objectives of 6E10 (green), synaptophysin (red) stained CA1 and cortex of 12- and 16-months old AD and AD/cKO. **H.** The graphs show the percentage loss of synaptophysin measured by Imaris rendering at 3 plaque niches from 3 sections in CA1 and cortex (*n*=four), *P<0.05. mo = month.

**Figure 3:**
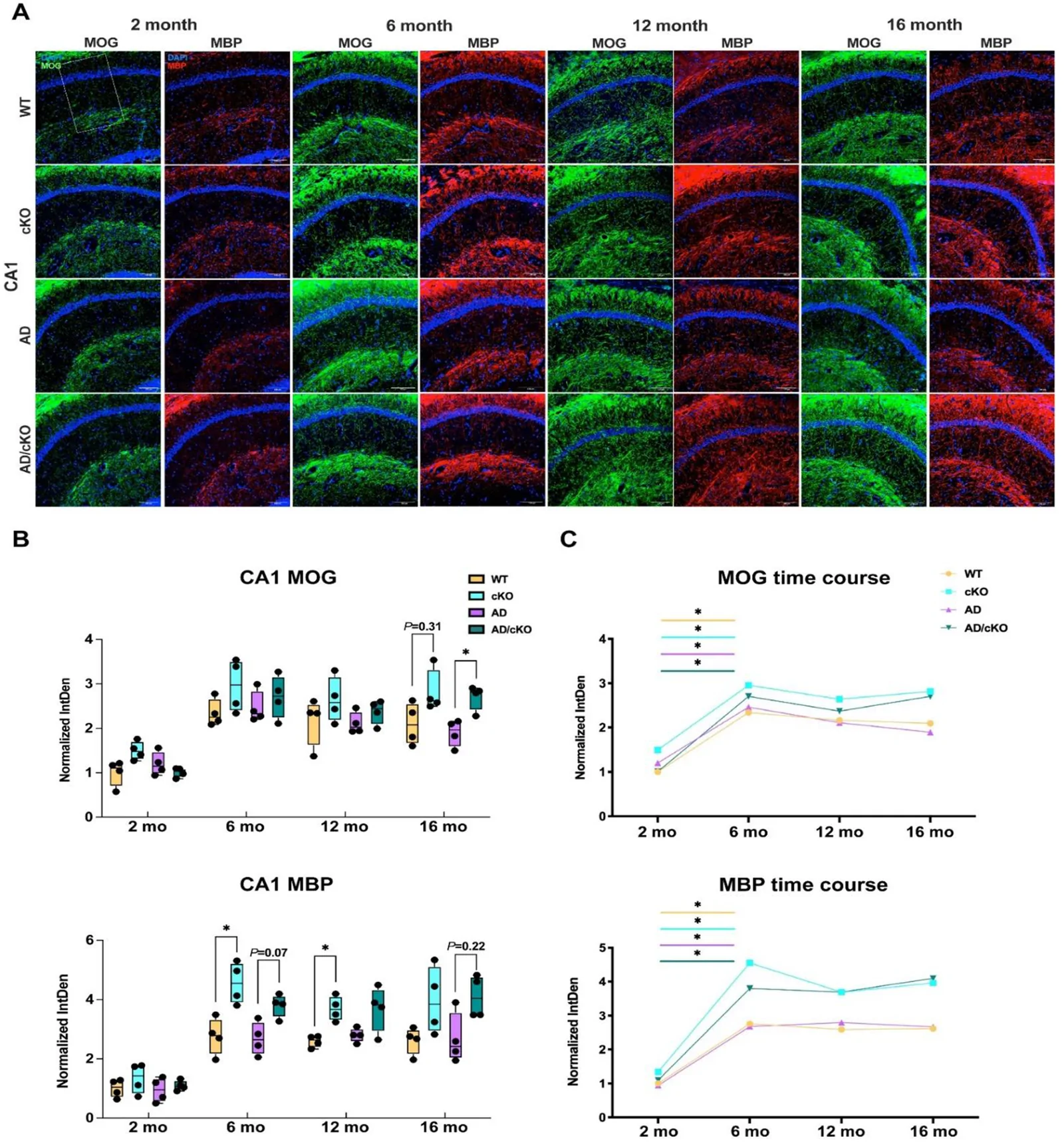
*Fus* depletion dependent increase in myelin profiles in AD/cKO hippocampal CA1. **A**. Representative 20X confocal images of MOG and MBP stained CA1 subregion of WT, cKO, AD, AD/cKO at 2, 6, 12 and 16 months. **B.** The graphs show the normalized density (MBP and MOG density at 2 months of age is set as 1) of MOG and MBP in the FOV of 3 sections in CA1 of 2-, 6-, 12- and 16-months old WT, cKO, AD, AD/cKO (*n*=four). *P<0.05, actual p value is shown for non-significant and trending down comparisons. **C**. Line plot shows the temporal course of normalized density of MOG and MBP at 2, 6, 12 and 16 months of age in WT, cKO, AD and AD/cKO. mo = month.

**Figure 4:**
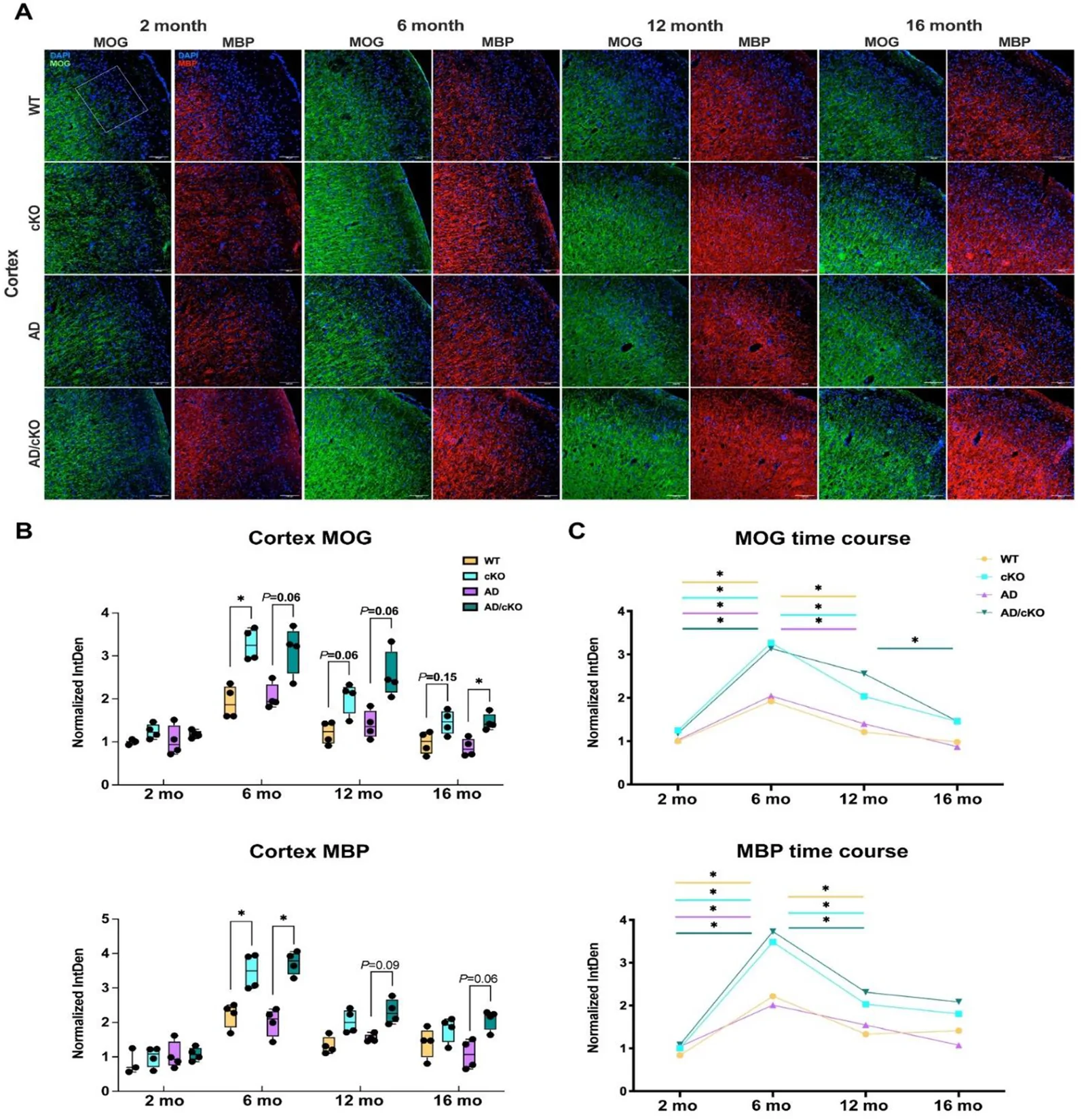
Fus-dependent increase in adaptive myelination profiles in the AD/cKO cortex. **A**. Representative 20X confocal images of MOG and MBP stained cortex L2-L3 of WT, cKO, AD, AD/cKO at 2, 6, 12 and 16 months of age. **B.** The graphs show the normalized density of MOG and MBP in the cortex L2-L3 ROI of 3 sections in 2, 6-, 12- and 16-months old WT, cKO, AD, AD/cKO (*n*=four). *P<0.05, ns= non-significant, actual *P* value is shown for trending up comparisons. **C**. Line plot shows the temporal course of normalized density of MOG and MBP at 2, 6, 12 and 16 months of age in WT, cKO, AD and AD/cKO. mo = month.

**Figure 5:**
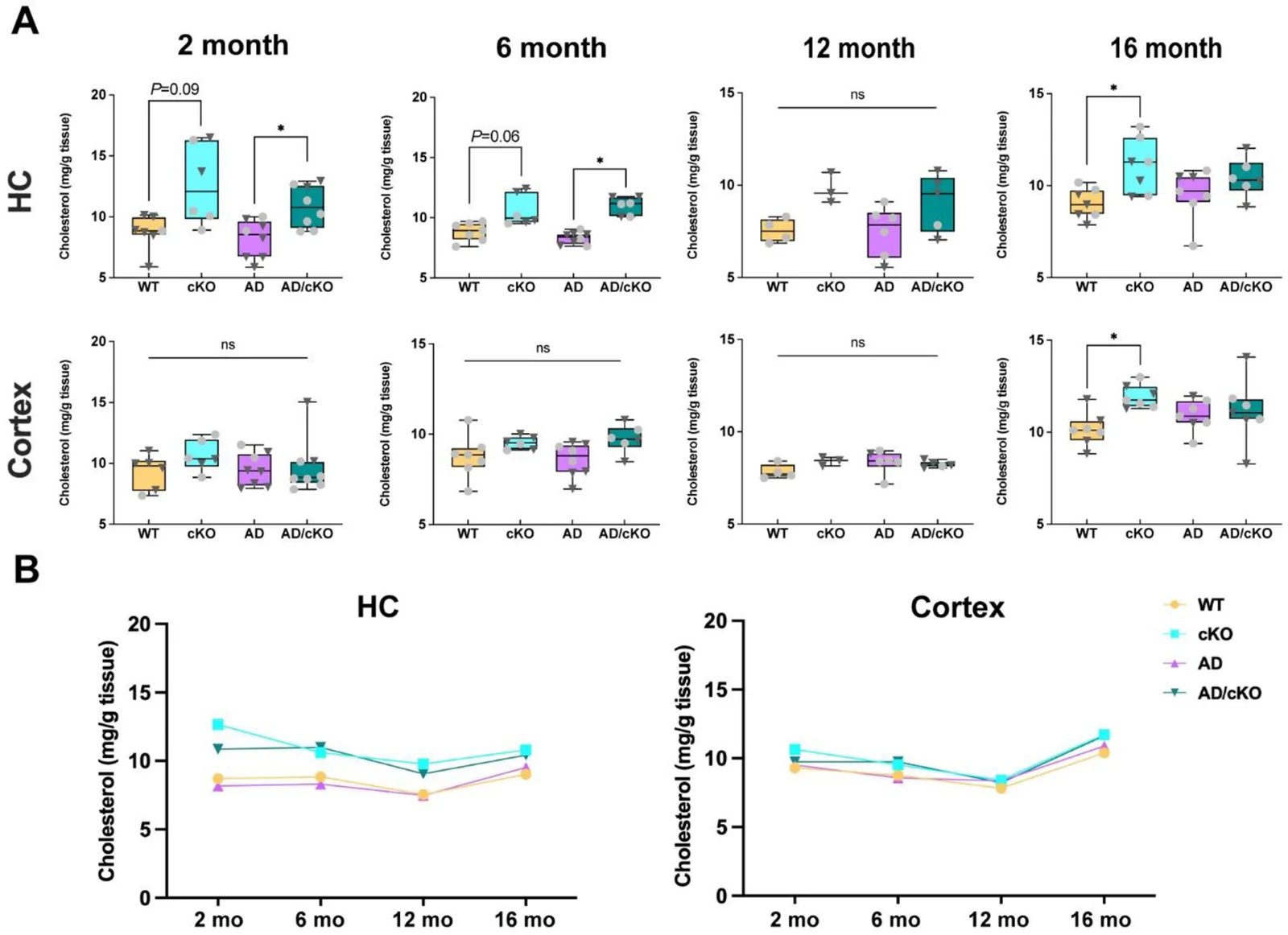
Cholesterol content is increased in the cKO and AD/cKO hippocampus. **A.** The graphs show the cholesterol content (mg/g of wet tissue) in hippocampi and cortices collected from 2-, 6-, 12- and 16-months old WT, cKO, AD and AD/cKO brains, * *P*<0.05, ns= non-significant, actual p value is shown for trending up comparisons. **B.** Line plot shows the temporal course of the cholesterol content (mg/g of wet tissue) from hippocampi and cortex collected from 2-, 6-, 12- and 16-months old WT, cKO, AD and AD/cKO. mo = month.

**Figure 6:**
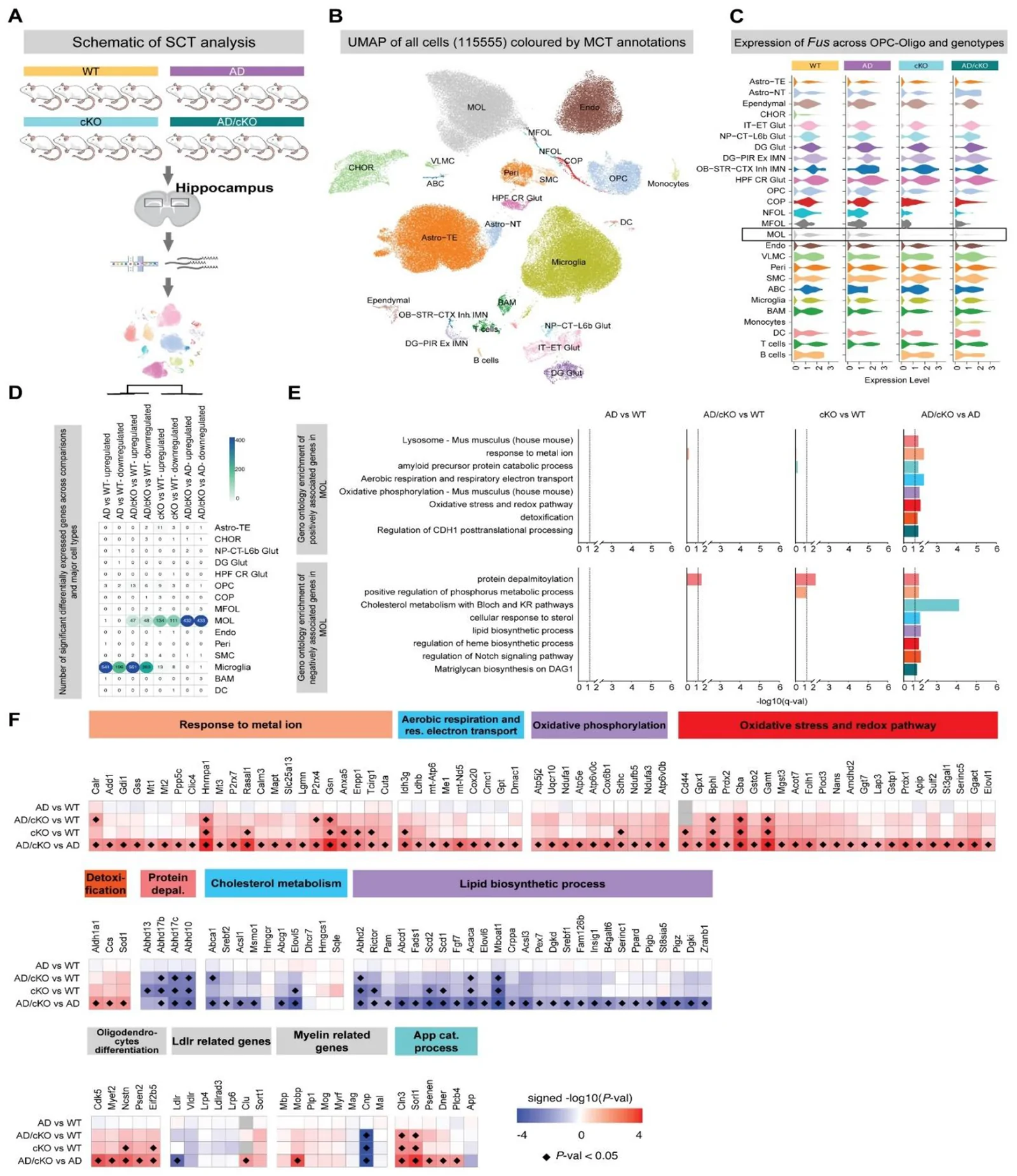
Mature oligodendrocytes exhibit pronounced transcriptomic alterations in AD/cKO compared with AD. **A.** Overview of the experimental design of SCT of hippocampi of WT, cKO, AD, and AD/cKO mice. **B.** UMAP visualization of the major cell populations identified by scRNA-seq. **C.** Violin plots showing *Fus* expression across major cell types and genotypes. **D.** Heatmap displaying the number of differentially expressed genes across genotype comparisons within each cell type. **E.** Gene ontology enrichment analysis for mature oligodendrocytes (MOL): the top panel highlights GO terms enriched among genes upregulated across genotype comparisons, while the bottom panel highlights GO terms enriched among downregulated genes. **F.** Heatmap showing the association of selected genes related to enriched Gene ontologies across genotype comparisons.

**Figure 7:**
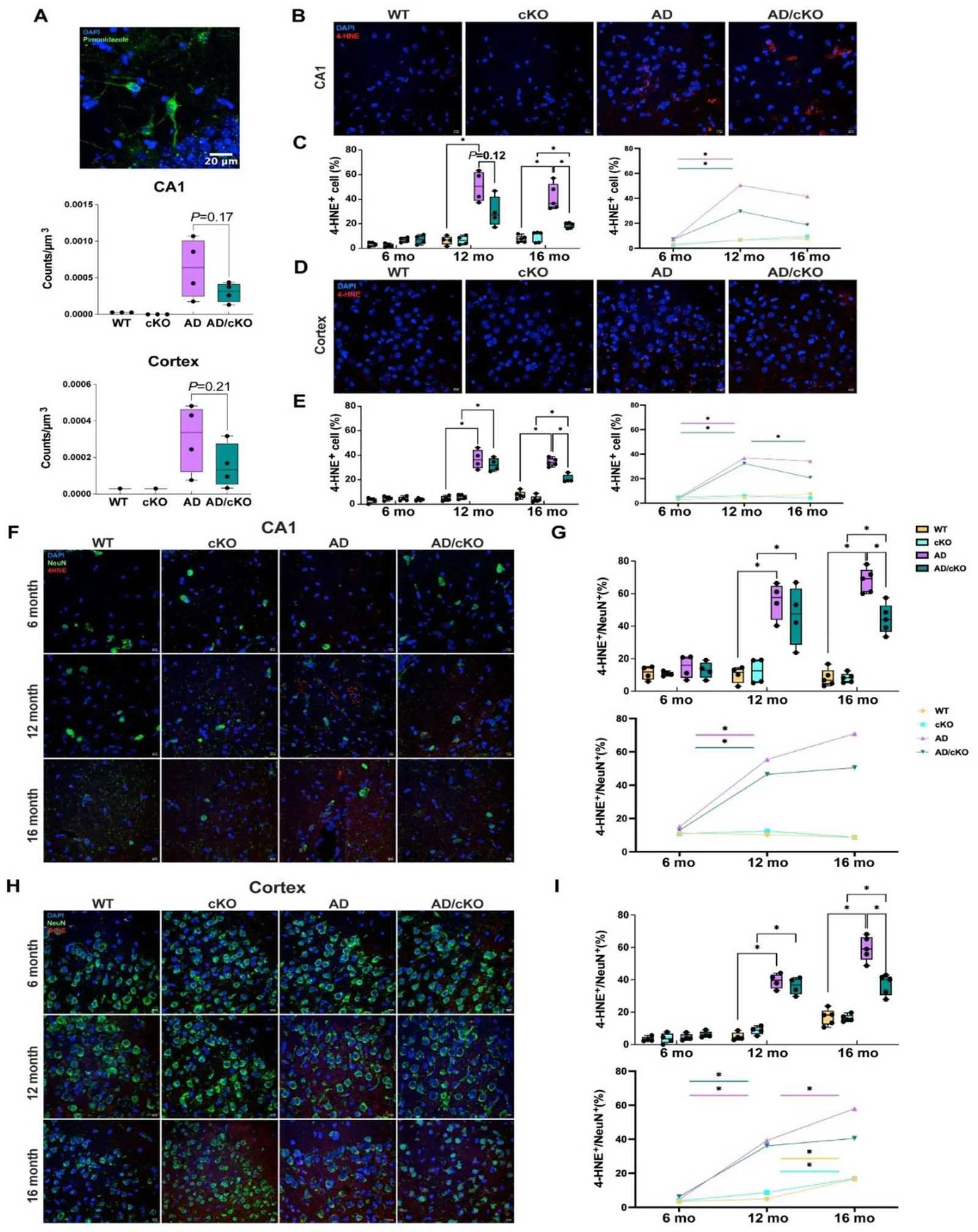
Oxidative damage measured by lipid peroxidation is reduced in the CA1 and cortex of the aged AD/cKO. **A.** Representative 20X confocal image of hippocampus neurons stained with an Ab to pimonidazole SH adducts of 4 months old AD. The graphs show the number of hypoxic neurons/μm^3^ in the CA1 and cortex in the FOV of 3 sections of WT, cKO (*n*=two), AD and AD/cKO (*n*=four), actual p value is shown for non-significant and trending down comparisons. **B.** Representative 40X confocal images of 16-months old WT, cKO, AD and AD/cKO CA1 stained with DAPI and an Ab for 4-HNE. **C**. The graphs show the 4-HNE+/DAPI+ cells (%) in the FOV of 3 sections imaged at 20X (*n*=five), * *P*<0.05. Line plot shows the temporal course of the percentage of 4-HNE+/DAPI+ cells at 6, 12 and 16 months of age in WT, cKO, AD and AD/cKO. **D.** Representative 40X confocal images of 16-months old WT, cKO, AD and AD/cKO cortex stained with DAPI and an Ab for 4-HNE. **E.** The graphs show the 4-HNE+/DAPI+ cells (%) in the FOV of 3 sections imaged at 20X (*n*=five), * *P*<0.05. Line plot shows the temporal course of the 4-HNE+/DAPI+ cell density at 6, 12 and 16 months of age in WT, cKO, AD and AD/cKO. **F.** Representative 40X confocal images of 6-, 12- and 16-months old WT, cKO, AD and AD/cKO CA1 stained with an Ab to NeuN and an Ab to 4-HNE. **G.** The graphs show the 4-HNE+/NeuN+ cells (%) in the FOV of 3 sections imaged at 20X (*n*=five), * *P*<0.05. Line plot shows the temporal course of the 4-HNE+/NeuN+ cells at 6, 12 and 16 months of age in WT, cKO, AD and AD/cKO. **H.** Representative 40X confocal images of 6-, 12- and 16-months old WT, cKO, AD and AD/cKO cortex stained with Abs to NeuN and 4-HNE. **I.** The graphs show the 4-HNE+/NeuN+ cells in 3 FOVs of 3 sections imaged at 20X (*n*=five 16 months and *n*=four 12 months), * *P*<0.05. Line plot shows the temporal course of the 4-HNE+/NeuN+ cells at 6, 12 and 16 months of age in WT, cKO, AD and AD/cKO. mo = month.

## Data Availability

Raw and processed data will be made available in GEO. The code and results for the differential expression analyses are available on GitHub: https://github.com/sudhagarbabu/Fus_cKO.
