# Supplementary material for: Fus-depleted oligodendrocytes reduce neuronal damage and Alzheimer’s disease progression in the AppNL-G-F mouse
Source: Brain. Author manuscript; Available in PMC 2026 Nov 3. (PMC13730162; doi:10.1093/brain/awag234)
Supplement: Supplementary file 1 [file NIHMS2214114-supplement-supplementary_zip.zip › brain-2025-03929-File009.pdf]

Supplementary Method

Generation of the *App*$^{NL\text{-}G\text{-}F}$/*Fus*^{OL}cKO line and Genotyping

The homozygous *App*$^{NL\text{-}G\text{-}F}$ mouse line (a kind gift of Dr. Saido[1]) was crossed with the *Fus*$^{fl/fl/CNPcre/+}$ line [2] to generate the *App*$^{NL\text{-}G\text{-}F}$/*Fus*$^{fl/fl/CNPcre/+}$. The new line was maintained by breeding *App*$^{NL\text{-}G\text{-}F}$/*Fus*$^{fl/fl/CNPcre/+}$ with the littermates *App*$^{NL\text{-}G\text{-}F}$/*Fus*$^{fl/fl/CNP+/+}$. For simplicity, we will refer in the text to *App*$^{NL\text{-}G\text{-}F}$/*Fus*^{OL}*cKO* as AD/cKO and *App*$^{NL\text{-}G\text{-}F}$/*Fus*$^{fl/fl/CNP+/+}$ as AD. The breeding of *Fus*$^{fl/fl/CNPcre/+}$ with the *Fus*$^{fl/fl}$ littermates [2] generated the *Fus*^{OL}cKO referred to as cKO and *Fus*$^{fl/fl}$ as WT. All mice were bred and housed in a barrier facility with a 12-hour light/dark cycle. Food and water were available ad libitum. Mouse genotypes were determined by PCR of ear snip-derived DNA following published protocols. [1,2] All efforts were made to minimize animal suffering and the number of animals used. The use of animals in this study followed the guidelines of the Institutional Animal Care and Use Committees (IACUC) of the University of Pittsburgh (Animal Welfare Assurance number D16-00118) and was approved by IACUC under protocol # 24034725.

Mouse Behavioural testing

Novel Spatial Recognition

To assess short-term recognition memory, we used the novel spatial recognition test which is similar to the novel object recognition but replaces the use of objects with visual cues. [3,4] After acclimation, mice were placed within a three-arm Y-shaped maze, in which one of the arms was inaccessible, surrounded by a black floor-to-ceiling curtain to minimize visual cues outside the maze. Mice were allowed to freely explore for 10 minutes, returned to their home cage and after 10 min prior were placed back in the three-arm maze with the barricade removed and allowed to freely explore for 5 minutes. The amount of time spent in each arm and the number of entries into each arm were tracked (EthoVision XT 9.0, Noldus Information Technology, Wageningen, Netherlands), exported for the analysis. The total entries are the sum of entries into each arm. The cumulative time spent in each arm, the percent duration in each arm was calculated as: $Duration\ in\ arm\ X\ (\%) = \frac{Time\ spent\ in\ arm\ X}{Sum\ of\ time\ spent\ in\ all\ arms} \times\ 100$. The preference in spending time in the novel arm relative to the familiar or start arms is the measure of short-term memory.

Spontaneous Alternation

To assess spatial working memory and exploration, we tested mice in the Y Maze.[5-7] In a maze with multiple arms, mice exhibit the spontaneous behaviour of alternating between arm choices with a greater frequency than re-entering the same arm most recently visited.[7] After acclimation, the mouse was placed at the end of the start arms of the maze facing the maze center and allowed to explore freely for 8 min. Behavioural tracking software (EthoVision XT 9.0, Noldus Information Technology, Wageningen, Netherlands) was used to automatically track the behaviour. From the sequence of arm entries, the number of alternation opportunities were counted as the total number of correct and incorrect triads. The percent alternation is the ratio of correct triads to the number of alternation opportunities: $Alternation\ (\%) = \frac{Correct\ triads}{Total\ opportunities} \times 100$. The correct alternation when the mouse entered three different arms consecutively was taken as a measure of spatial working memory.[3]

Elevated Plus Maze (EPM)

To assess anxiety behaviour we used the elevated plus maze.[8] Mice were acclimated for 30 minutes prior to testing in a well-lit room. The mouse was placed in a closed arm of the EPM) which has two closed arms and two open arms and was allowed to explore the apparatus for 5 min. Experiments were conducted at full light. All movements were recorded by an overhead camera using SMART 3.0, Panlab S.I. All the videos were collected and automatically analysed by behaviour tracking system (SMART 3.0, Panlab S.I., Barcelona, Spain). We measured time spent in the open arms as a measure of anxiety and the total distance traveled, as a measure of activity. Time spent in the open arms was taken as an inverse measure of anxiety.

Single-Cell Transcriptomics (SCT)

Cell suspension preparation and single-cell (sc)RNA-seq library generation

Mice were deeply anesthetized and perfused intracardially with ice cold PBS, brains were removed, and hippocampi were quickly dissected on ice. We collected hippocampi from 2 brains/sample. The cell suspensions were prepared using the Adult Brain Dissociation Kit

(Miltenyi Biotec 130-107-677) according to the manufacturer's instructions with the following modifications that increased cell yield. The tissues were chopped into small pieces on ice prior to adding to the Enzyme 1 mix, after enzymatic and mechanical dissociation, the mixtures were triturated with P1000 5-10 times to dissociate undigested pieces of tissue, red blood cell removal step was omitted since brains were thoroughly perfused with PBS. Cell counts and viability were measured with AO/PI staining in an automated cell count system (Cellometer Cell Viability Counter, Nexcelom). Cells were immediately fixed using 10X FLEX reagents and 10x Genomics protocol, counted post-fixation and stored at -80 C according to 10X Genomics protocol. Four biological replicates per genotype (M:F ratio was 40:60), each replicate containing hippocampi from two brains, were submitted to the Single Cell Transcriptomics Core at the University of Pittsburgh. Each sample contained ~300,000 cells and the viability was >80%. Libraries were prepared using the Chromium Fixed RNA Profiling Reagents Kit for Multiplexed Samples (10x Genomics) according to the manufacturer's protocol. Libraries sequencing performed by the Health Sciences Sequencing Core at the University of Pittsburgh generated 1.8 billion reads which covers more than 10,000 reads per cell.

SCT data processing

Raw FASTQ sequencing files were demultiplexed and aligned to the Chromium Mouse Transcriptome Probe Set v1.0.1 (mm10-2020A) from 10x Genomics using the Cell Ranger multi pipeline (version 8.0.1). The resulting filtered feature-barcode matrices were imported and merged using the Scanpy Python toolkit [9] for downstream analysis. Initial quality control (QC) involved filtering out low-quality cells based on gene detection thresholds. Specifically, cells with fewer than 350 detected genes (n_genes_by_counts) were excluded, with this threshold determined from the distribution of gene counts across all cells. In addition, cells exhibiting high mitochondrial gene expression—defined as exceeding five median absolute deviations (MADs) above the median—were removed to eliminate stressed or dying cells. To further improve data quality, potential doublets were identified and excluded using the scDblFinder function from the scDblFinder package (version 1.20.2),[10] with the parameter dbr.sd set to 1. All cells flagged as doublets were removed from subsequent analyses.

SCT integration and clustering

For dataset integration, we employed the scVI pipeline using an iterative approach to enhance cluster resolution and eliminate technical artifacts.[11] In the first iteration, the top 2000 highly variable genes were selected using the sc.pp.highly_variable_genes function with the flavor parameter set to "seurat_v3". The scVI model was then trained using sample as a categorical covariate and n_genes_by_counts and pct_counts_mt as a continuous covariate. Clustering was performed using the Leiden algorithm with a resolution of 1.0, followed by a second round of subclustering using the same parameters to increase granularity. After each clustering round, we calculated cluster-level mean QC metrics including the number of genes per cell, total UMI counts, mitochondrial gene percentage, and scDblFinder-assigned doublet scores. Clusters exhibiting high mitochondrial content—defined as exceeding the 99th percentile across all clusters—or mean doublet scores above 0.1 were flagged and removed. This iterative refinement was performed twice to exclude low-quality or artifactual clusters. However, biologically relevant intermediate states, such as those representing oligodendrocyte precursor cell (OPC) to oligodendrocyte (Oligo) lineages, which were sometimes flagged as doublets, were retained. To better resolve microglial and mature oligodendrocyte (MOL) subpopulations, we used the subclusters derived from the second iteration as the starting point. Clusters lacking distinct marker gene expression profiles were systematically merged using the *merge_clusters* function from the transcriptomic clustering package (https://github.com/AllenInstitute/transcriptomic_clustering), which implements the split-and-merge strategy.[12] To identify marker genes specific to each cluster, we used the FindAllMarkers function[13] implemented in Seurat V5 with the parameters: only.pos = TRUE, min.pct = 0.25, logfc.threshold = 0.25, and max.cells.per.ident = 500.

Cell type annotation

Cell type annotation was conducted using the MapMyCells platform (https://knowledge.brain-map.org/mapmycells/process/), which enables reference-based classification of single-cell transcriptomic data. As a reference taxonomy, we employed the 10x Genomics Whole Mouse Brain dataset. Annotation was performed using the Hierarchical Mapping algorithm, which assigns cell identities across multiple hierarchical levels of brain cell types. For major cell type (MCT) classification, final identities were determined by integrating annotations across these hierarchical levels provided by MapMyCells. At the high-resolution cell type (HCT) level, we further refined annotations by incorporating curated subclusters of microglia and mature oligodendrocytes

(MOLs). This refinement was guided by a split-and-merge clustering strategy, expression profiles of canonical marker genes, and distinct transcriptional signatures associated with individual clusters.

Ambient RNA removal

Potential ambient RNA contamination was removed using decontX,[14] and the corrected expression matrix was subsequently used for downstream differential gene expression analyses and marker gene identification.

Differential Gene Expression and Functional Gene Ontology Enrichment Analysis

Differential gene expression (DGE) analysis was performed using a pseudobulk-based approach implemented in muscat (v.1.20.0),[15] employing the default edgeR method. Raw counts were aggregated at the cell type level to generate pseudobulks across celltypes. DGE analysis was conducted by comparing genotypes (experimental groups), while controlling for age and sex as covariates. Genes identified as significantly differentially expressed were subsequently used for functional enrichment analysis. Gene Ontology (GO) enrichment was carried out using the Metascape [16]online tool.

Cell type composition analysis

Cell fractions were calculated for each sample. Cell types with fewer than 20 cells per experimental group were excluded from the analysis. A generalized linear model (GLM) was fitted using the glm function in R, with age and sex as covariates. *P*-values were adjusted using the Benjamini-Hochberg (BH) method via the *P*.adjust function, treating cell type as a factor to account for multiple testing across cell types.

Biochemical Studies: Protein Isolation and Western Blot

Mice were deeply anesthetized, perfused with cold PBS and rapidly decapitated. Brains were removed and prefrontal cortices and hippocampi were carefully dissected out on ice.[17] All tissues were rapidly frozen in liquid nitrogen and stored at -80C°. Frozen tissue was placed into a 1.5mL

tube containing homogenization buffer (25mM Tris-HCL pH 7.4, 1mM EDTA, 150mM NaCl, 1% SDS, Protease (Complete Mini, EDTA free, Roche, #11836170001) and Phosphatase inhibitors (PosSTOP, Roche, #04906837001)), and disrupted with a motorized pestle, then further sonicated briefly. Samples were centrifuged at 12,000 rpm for 20 minutes at 4°C and supernatant was collected. Extracted proteins concentrations were determined using a Pierce BCA Protein Assay Kit (Thermo Fisher, Waltham, Massachusetts) and stored at 80°C. Proteins were separated in 4-12% gradient Bis-Tris gels (NuPage) by SDS-PAGE, transferred to Immobilon FL membranes and reacted with primary Ab PLP (AA3 Rat Mab), MBP (Mouse Mab, Biolegend, # 808402) and α-tubulin (Mouse Mab, Sigma-Aldrich, #T5168) and labeled with IR Dye 680RD/800CW secondary's (Li-Cor Biosciences). Membranes were imaged using Odyssey Image Studio software and quantified in FIJI. Loading accuracy was determined using αtubulin as internal control.

Imaging

PLP was imaged using the Keyence BZ-X810 microscope,1 FOV was imaged at 20x in the somatosensory cortex. Tiled images were taken with an autofocus set for 5 z-slices over 29.9um per image and brightness and black balance of each channel kept the same per section, then aligned per channel and stitched together using the BZ-X800 Imaging software.

Imaging Data Analysis

Images were analysed using Fiji with PLP intensity calculated using 5 equal sized ROIs extending over layers 2 and 3 of the cortex.

For C3+ astrocytes, DAPI, S100β were rendered by Surface function and adjusted with the quality filter to best match the confocal image. C3 was rendered using the spot feature with an estimated diameter of 4 µm and C3+/S100β+ cells were identified by filtering with the distance of the spot to the nucleus set at 3 µm. A similar workflow was established for C3+/GFAP+.

Linear Mixed-Effects Model

To evaluate the longitudinal changes between 6 and 12 months of age, a linear mixed-effects (LME) model was fitted with age (categorical: 6,12), genotype (WT, cKO, AD, ADcKO), and

their interaction (Age x Genotype) as fixed effects, and a random intercept per animal to account for repeated measures: [18]

$$Behavioural\ Response \sim Age\ x\ Genotype + (1|AnimalID)$$

The three effects (age, genotype, and their interaction) were compared by likelihood ratio comparison of nested models, with Holm-Bonferroni correction applied across all three tests ($\alpha = 0.05$). Genotype-specific slopes (6-to-12-month change) were derived from the age and interaction coefficients, and all pairwise slope differences were tested using approximate t-tests with Holm-Bonferroni correction across the six comparisons.

We performed cross-sectional analyses on the independent measurements at 16 months. At each timepoint (6, 12, and 16 months), genotype effects were evaluated using Welch's one-way ANOVA to accommodate unequal group variances, with the three omnibus p-values corrected jointly using the Holm-Bonferroni method. Normality was assessed per group using the Shapiro-Wilk test, and homogeneity of variance was evaluated with Levene's test. Where the omnibus test was significant, pairwise post-hoc comparisons were performed using the Games-Howell test, followed by Holm-Bonferroni correction within each timepoint. Within-genotype age effects were similarly tested using Welch's ANOVA across the three timepoints, with Games-Howell pairwise comparisons (6 vs 16 and 12 vs 16 months) and Holm-Bonferroni correction applied within each genotype. All statistical approaches for behavioural responses were analyzed using a customized MATLAB script.

References

1. Saito T, Matsuba Y, Mihira N, et al. Single App knock-in mouse models of Alzheimer's disease. *Nat Neurosci*. May 2014;17(5):661-3. doi:10.1038/nn.3697
2. Guzman KM, Brink LE, Rodriguez-Bey G, et al. Conditional depletion of Fus in oligodendrocytes leads to motor hyperactivity and increased myelin deposition associated with Akt and cholesterol activation. *Glia*. Mar 18 2020;doi:10.1002/glia.23825
3. Sukoff Rizzo SJ, Silverman JL. Methodological Considerations for Optimizing and Validating Behavioral Assays. *Curr Protoc Mouse Biol*. Dec 1 2016;6(4):364-379. doi:10.1002/cpmo.17
4. Sukoff Rizzo SJ, Anderson LC, Green TL, McGarr T, Wells G, Winter SS. Assessing Healthspan and Lifespan Measures in Aging Mice: Optimization of Testing Protocols, Replicability, and Rater Reliability. *Curr Protoc Mouse Biol*. Jun 2018;8(2):e45. doi:10.1002/cpmo.45
5. Tucker LB, Fu AH, McCabe JT. Performance of Male and Female C57BL/6J Mice on Motor and Cognitive Tasks Commonly Used in Pre-Clinical Traumatic Brain Injury Research. Research Support, Non-U.S. Gov't. *Journal of neurotrauma*. May 1 2016;33(9):880-94. doi:10.1089/neu.2015.3977
6. Bailey KR, Crawley JN. Anxiety-Related Behaviors in Mice. In: nd, Buccafusco JJ, eds. *Methods of Behavior Analysis in Neuroscience*. 2009. *Frontiers in Neuroscience*.
7. Hughes RN. The value of spontaneous alternation behavior (SAB) as a test of retention in pharmacological investigations of memory. *Neuroscience and biobehavioral reviews*. Sep 2004;28(5):497-505. doi:10.1016/j.neubiorev.2004.06.006
8. Pellow S, Chopin P, File SE, Briley M. Validation of open:closed arm entries in an elevated plus-maze as a measure of anxiety in the rat. Comparative Study. *Journal of neuroscience methods*. Aug 1985;14(3):149-67. doi:10.1016/0165-0270(85)90031-7
9. Wolf FA, Angerer P, Theis FJ. SCANPY: large-scale single-cell gene expression data analysis. *Genome biology*. Feb 6 2018;19(1):15. doi:10.1186/s13059-017-1382-0
10. Germain PL, Lun A, Garcia Meixide C, Macnair W, Robinson MD. Doublet identification in single-cell sequencing data using scDblFinder. *F1000Res*. 2021;10:979. doi:10.12688/f1000research.73600.2
11. Gayoso A, Lopez R, Xing G, et al. A Python library for probabilistic analysis of single-cell omics data. *Nat Biotechnol*. Feb 2022;40(2):163-166. doi:10.1038/s41587-021-01206-w
12. Jorstad NL, Song JHT, Exposito-Alonso D, et al. Comparative transcriptomics reveals human-specific cortical features. *Science*. Oct 13 2023;382(6667):eade9516. doi:10.1126/science.ade9516
13. Hao Y, Stuart T, Kowalski MH, et al. Dictionary learning for integrative, multimodal and scalable single-cell analysis. *Nat Biotechnol*. Feb 2024;42(2):293-304. doi:10.1038/s41587-023-01767-y
14. Yang S, Corbett SE, Koga Y, et al. Decontamination of ambient RNA in single-cell RNA-seq with DecontX. *Genome biology*. Mar 5 2020;21(1):57. doi:10.1186/s13059-020-1950-6
15. Crowell HL, Soneson C, Germain PL, et al. muscat detects subpopulation-specific state transitions from multi-sample multi-condition single-cell transcriptomics data. *Nature communications*. Nov 30 2020;11(1):6077. doi:10.1038/s41467-020-19894-4

16. Zhou Y, Zhou B, Pache L, et al. Metascape provides a biologist-oriented resource for the analysis of systems-level datasets. *Nature communications*. Apr 3 2019;10(1):1523. doi:10.1038/s41467-019-09234-6

17. Tung TH, Lai WD, Lee HC, Su KP, Panunggal B, Huang SY. Attenuation of Chronic Stress-Induced Depressive-like Symptoms by Fish Oil via Alleviating Neuroinflammation and Impaired Tryptophan Metabolism in Aging Rats. *J Agric Food Chem*. Oct 11 2023;71(40):14550-14561. doi:10.1021/acs.jafc.3c01784

18. Laird NM, Ware JH. Random-effects models for longitudinal data. *Biometrics*. Dec 1982;38(4):963-74.
