# Supplementary material for: Fus-depleted oligodendrocytes reduce neuronal damage and Alzheimer’s disease progression in the AppNL-G-F mouse
Source: Brain. Author manuscript; Available in PMC 2026 Nov 3. (PMC13730162; doi:10.1093/brain/awag234)
Supplement: Supplementary file 1 [file NIHMS2214114-supplement-supplementary_zip.zip › brain-2025-03929-File011.pdf]

Supplementary Video Legend

Supplementary Video 1. Synaptophysin quantification of AD using Imaris. The video shows 3D image of the fluorescently labeled synaptophysin puncta (red) and the plaque stained with 6E10 (green) in the FOV followed by Imaris rendering of synaptophysin puncta as spots in the FOV and the localization of the spots at the plaque niche. Video available at FigShare: https://doi.org/10.6084/m9.figshare.32671602

Supplementary Video 2. Synaptophysin quantification of AD/cKO using Imaris. The video shows that AD/cKO mice retain more synaptophysin puncta (red) and rendered synapses (grey spheres) within the plaque niche, demonstrating preservation of synapses. Video available at FigShare: https://doi.org/10.6084/m9.figshare.32682372
