# Supplementary material for: Fus-depleted oligodendrocytes reduce neuronal damage and Alzheimer’s disease progression in the AppNL-G-F mouse
Source: Brain. Author manuscript; Available in PMC 2026 Nov 3. (PMC13730162; doi:10.1093/brain/awag234)
Supplement: Supplementary file 1 [file NIHMS2214114-supplement-supplementary_zip.zip › brain-2025-03929-File012.pdf]

# Supplementary Figures

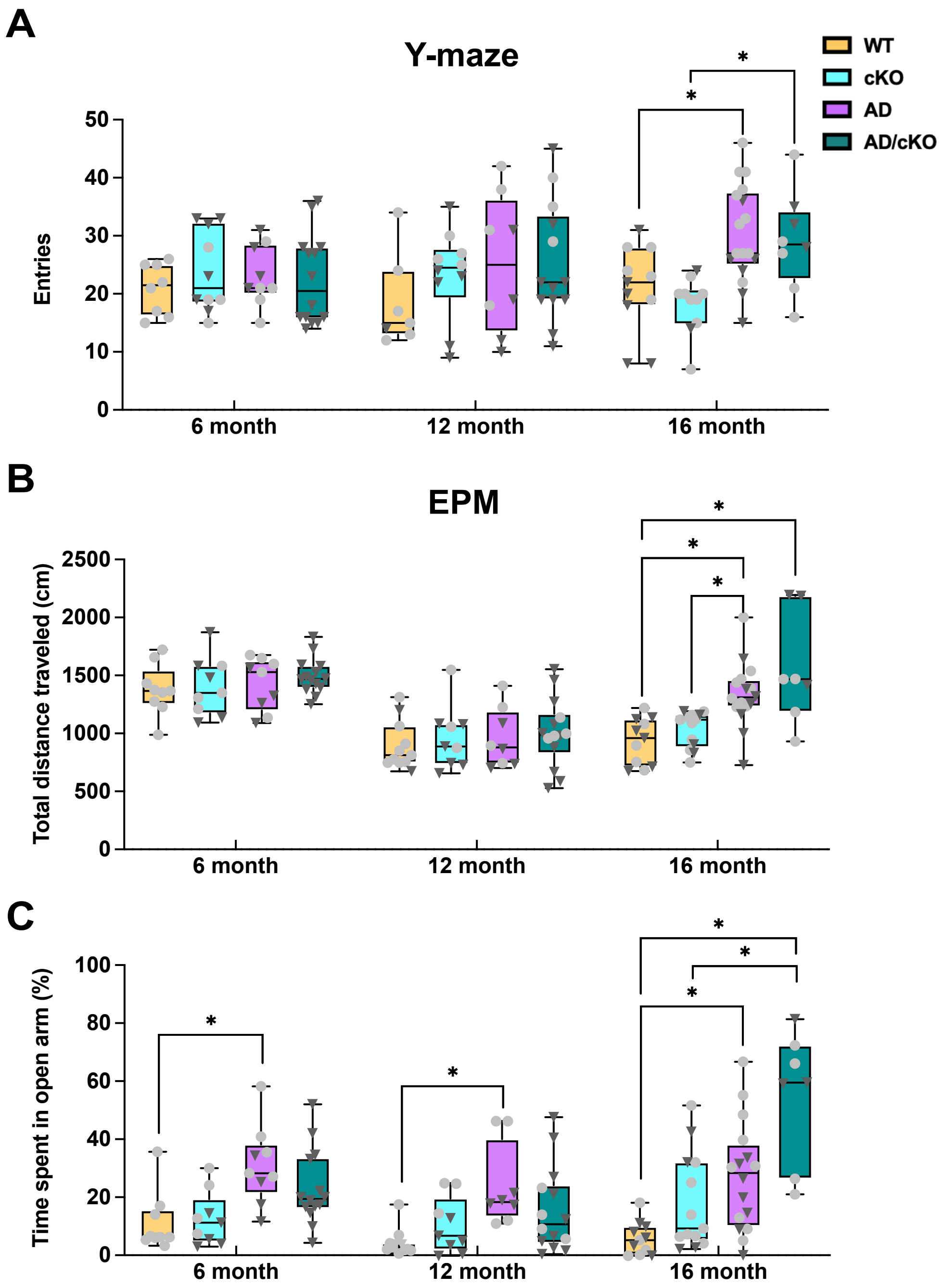


**Supplementary Figure 1. The AD and AD/cKO show similar anxiolytic behavior and hyperactivity. (A)** Graphs show the number of entries in the arms of the Y maze. The entries are statistically higher in the AD and AD/cKO vs WT and cKO at 16 months of age *$P$<0.05. **(B)** Graphs show the total distance travelled in the EPM. The distance is statistically higher in the AD and AD/cKO vs WT and cKO at 16 months of age * $P$<0.05. **(C)** Graphs show the time spent in the open arm of the EPM. The AD mice spent a statistically significant greater time in the open arm vs the WT at 6, 12 and 16 months of age. * $P$<0.05. The AD/cKO mice spent statistically significant greater time in the open arm at 16 months of age vs WT and cKO. * $P$<0.05.

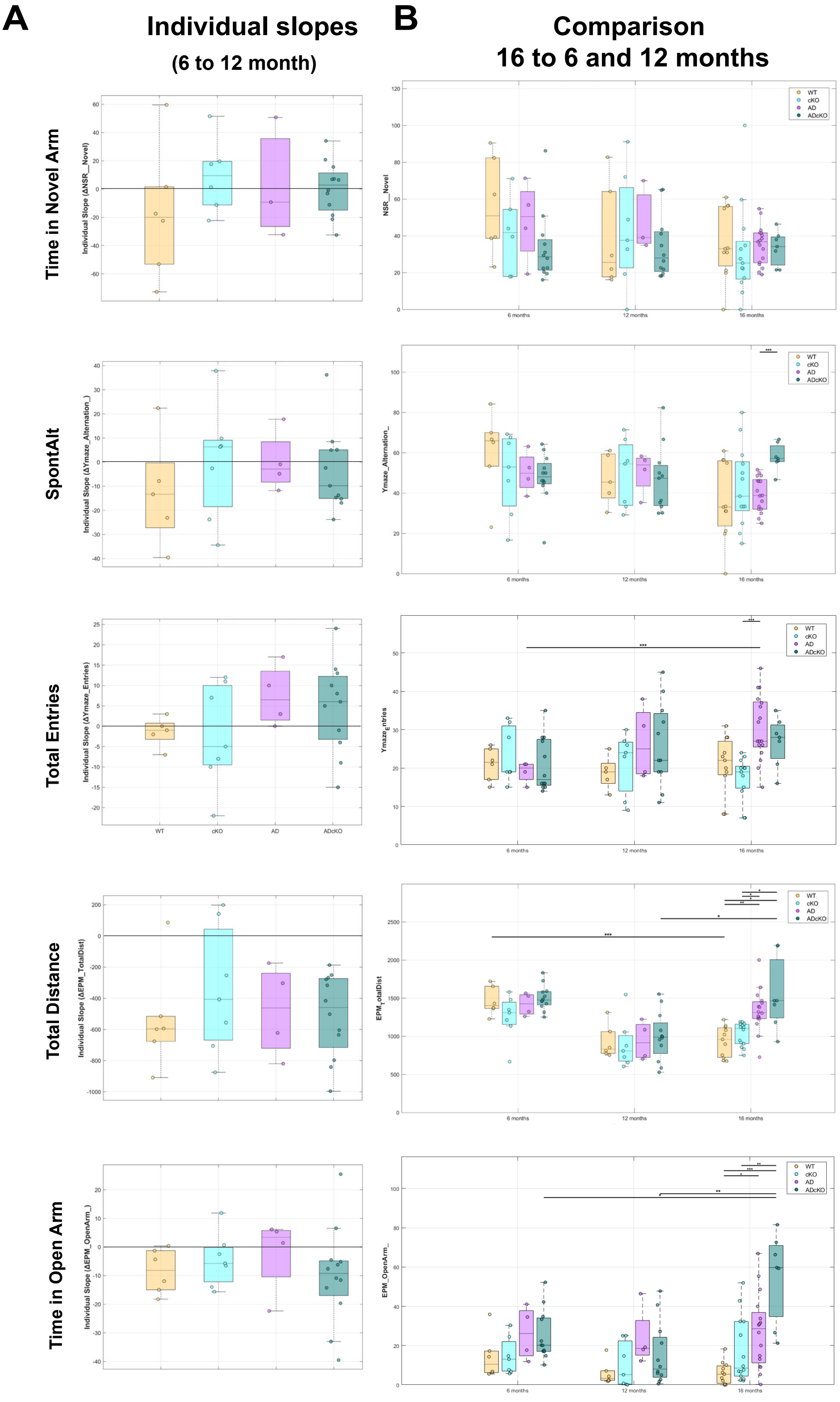


**Supplementary Figure 2. LME model analysis for 6 and 12 months age groups and Comparison of 16 months old group with the 6 and 12 months old cohorts. (A)** Slopes of subjects in each genotype show an age effect in the WT most prominent for the measures of hyperactivity (total entries and distance travelled). **(B)** Comparisons between 16 vs 6 and 16 vs 12 months. A genotype effect is present between 16 months AD/cKO and AD for spontaneous alternation. A genotype effect is present between 16 months AD and cKO for total entries. An age effect is present between AD 16 vs 6 months for total entries. A genotype effect is present for 16 months AD/cKO vs cKO and WT, AD vs cKO and WT for total distance. An age effect is present in the AD/cKO genotype at 16 vs 12 months and in the WT at the 16 vs 6 months comparison for total distance. A genotype effect is present for 16 months AD/cKO vs cKO and WT, AD vs WT for time in the open arm. An age effect is present in the AD/cKO genotype at 16 months vs. 12 and 6 months in the open arm. * *P*<0.05, ** *P*<0.01, *** *P*<0.001.

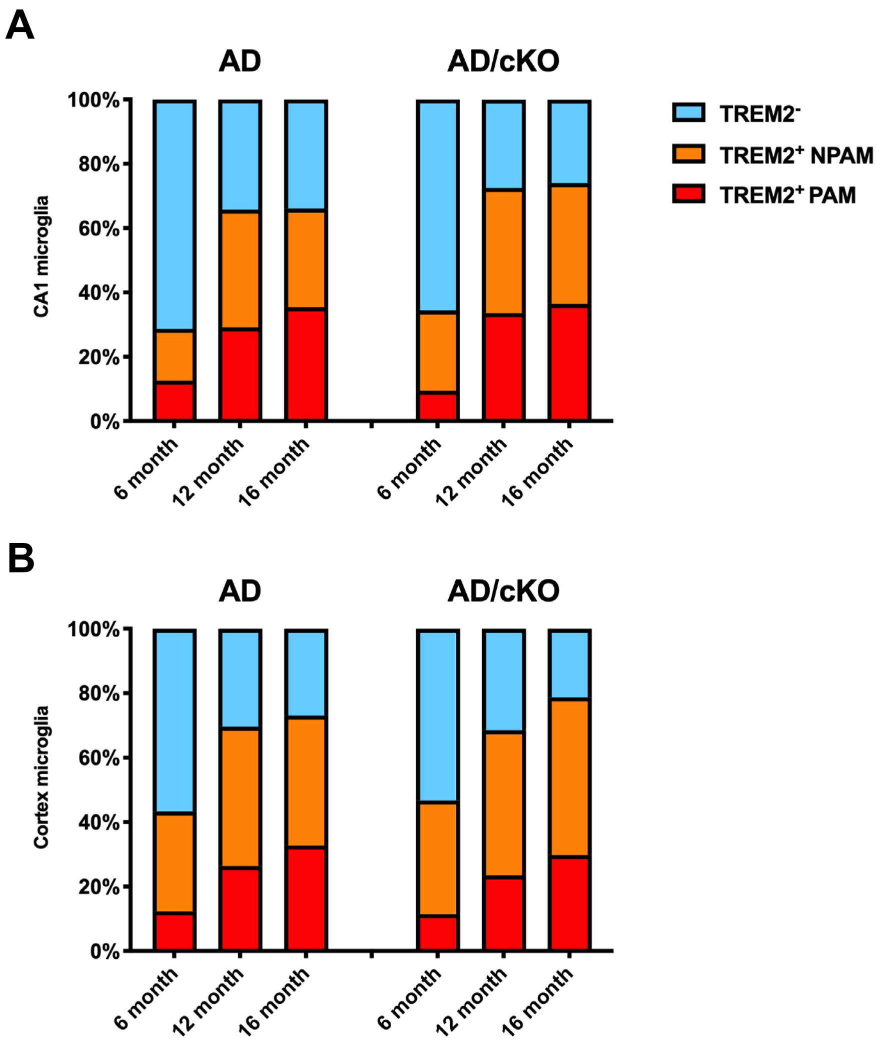


**Supplementary Figure 3. Total, Plaque Associated and Non-Plaque Associated TREM2+ microglia increase similarly in AD and AD/cKO. (A)** Stacked bar graphs show the relative proportion of TREM2+ PAM and NPAM and TREM2- microglia (see Methods) in CA1 at 6, 12 and 16 months. **(B)** Stacked bar graphs show the relative proportion of TREM2+ PAM and NPAM and TREM2- microglia in cortex at 6, 12 and 16 months. TREM2+ total, PAM and NPAM increase progressively from 6 to 16 months. There is no statistically significant difference between the AD and AD/cKO.

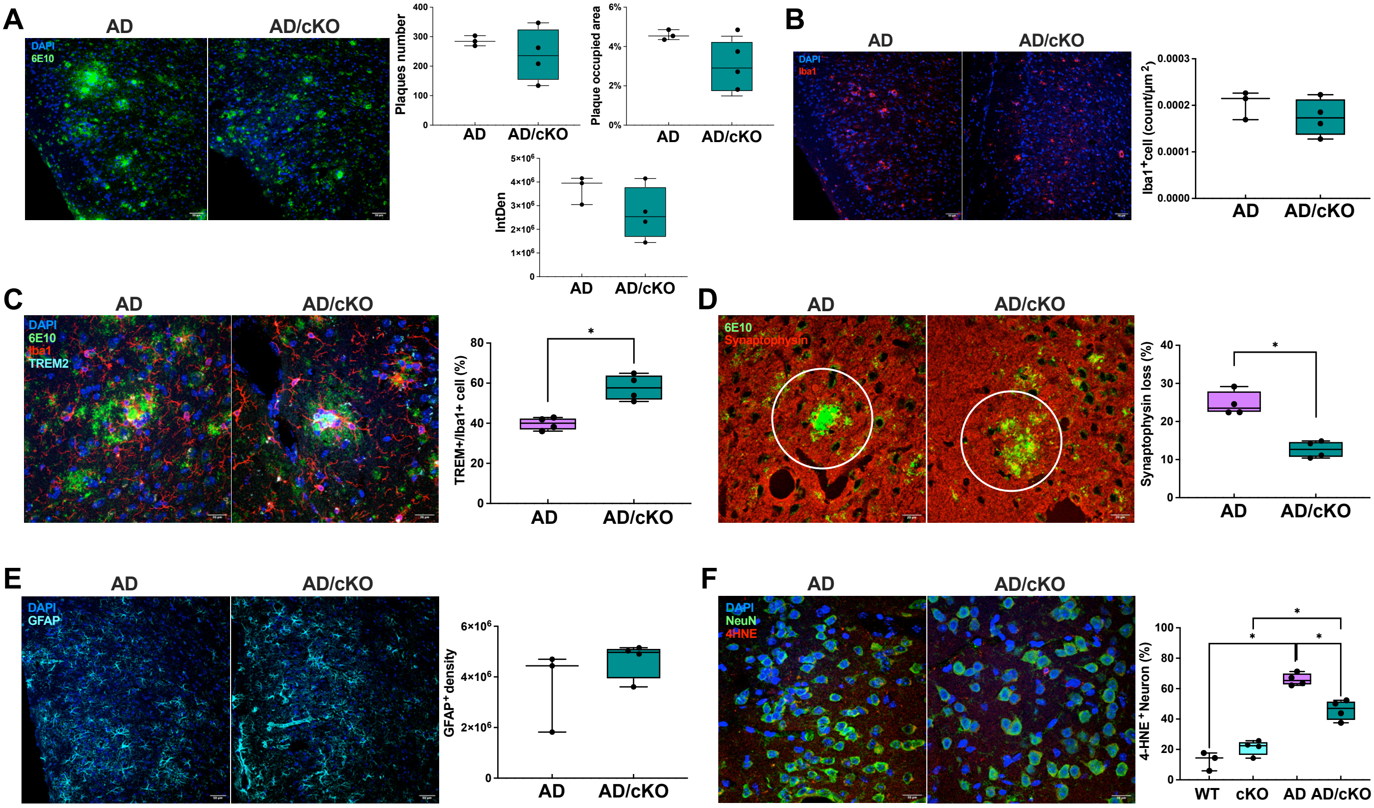


**Supplementary Figure 4. Plaque burden, microglia and astrocyte density, TREM2+ microglia at the plaque niche, 4-HNE+/NeuN+ cells in the mPFC. (A)** Representative 20X confocal images of cryosections stained with 6E10 to label β-amyloid and DAPI to identify nuclei in the mPFC of 16-months old AD and AD/cKO. Graphs show the plaque counts, the area occupied by the plaques and the integrated density (intensity of fluorescence per area) per FOV. There are no statistically significant differences. **(B)** Representative 20X confocal images of Iba1 stained cells in mPFC of 16-months old AD and AD/cKO. Graphs represent the Iba1+ cell counts in the FOV of 3 sections (*n*=four). There are no statistically significant differences. **(C)** Representative 40X confocal images of 6E10 (green), Iba1 (red) and TREM2 (cyan) stained mPFC cryosections of 16-months old AD and AD/cKO. Graphs represent the TREM2+/Iba1+(%) at 3 plaque niches from 3 sections (*n*=four). **(D)** Representative 40X confocal images of 6E10 (green), synaptophysin (red) stained mPFC cryosections of 16-months old AD and AD/cKO. Graphs represent the synaptophysin loss (%) measured by Imaris rendering at 3 plaque niches from 3 sections (*n*=four), $*P<0.05$. **(E)** Representative 20X confocal images of GFAP stained cells in the mPFC of 16-months old AD and AD/cKO. Graphs represent the GFAP+ profile density (AU) in the FOV of 3 sections (*n*=four). There are no statistically significant differences. **(F)** Representative 40X confocal images of 16-months old AD and AD/cKO mPFC cryosections stained with antibodies to NeuN and 4-HNE. Graphs represent the 4-HNE+/NeuN+ cells (%) in the FOV of 3 sections imaged at 20X (*n*=four), $* P<0.05$. Scale bar is 20µm.

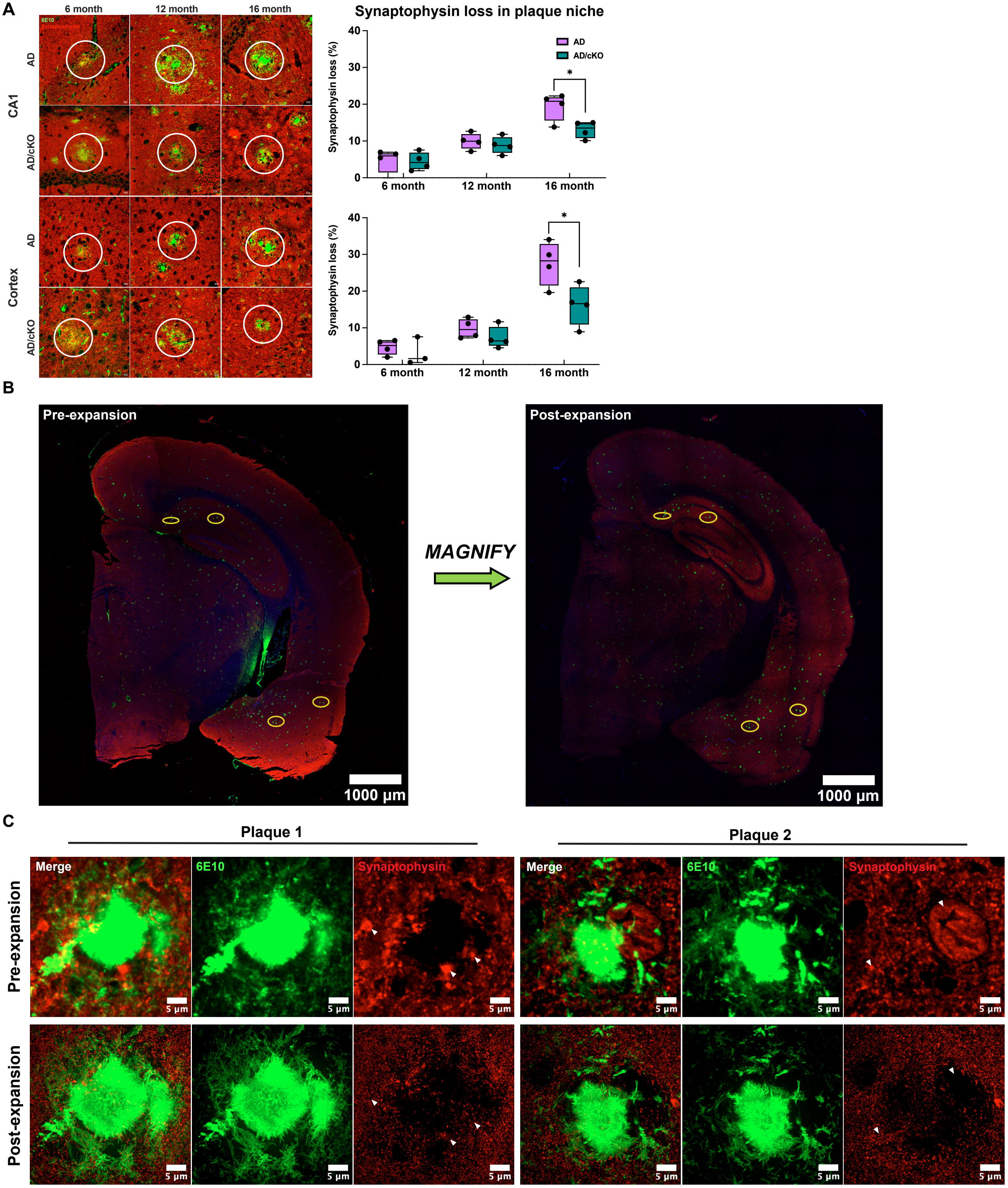


**Supplementary Figure 5. Enhanced resolution of synaptophysin puncta and reduced background noise with MAGNIFY. (A)** Representative 40X confocal images of 6E10 (green), synaptophysin (red) stained CA1 and cortex of 6-, 12- and 16-months old AD and AD/cKO. The graphs show the percentage loss of synaptophysin measured by Imaris rendering at 3 plaque niches from 3 sections in CA1 and cortex ($n$=four). Loss of synaptophysin at the plaque niche is reduced in the 16 months AD/cKO vs AD. $P<0.05$. **(B)** Pre- and post-expansion images taken with 4X objective of the AD brain hemisphere stained with 6E10 (green), synaptophysin (red). Circled in yellow are plaques sampled for comparison of pre- and post-images. **(C)** 60X images of 2 individual plaque niches stained with 6E10 (green), synaptophysin (red) pre-and post-expansion. There is enhanced resolution of synaptophysin puncta (arrowheads) and reduced background noise.

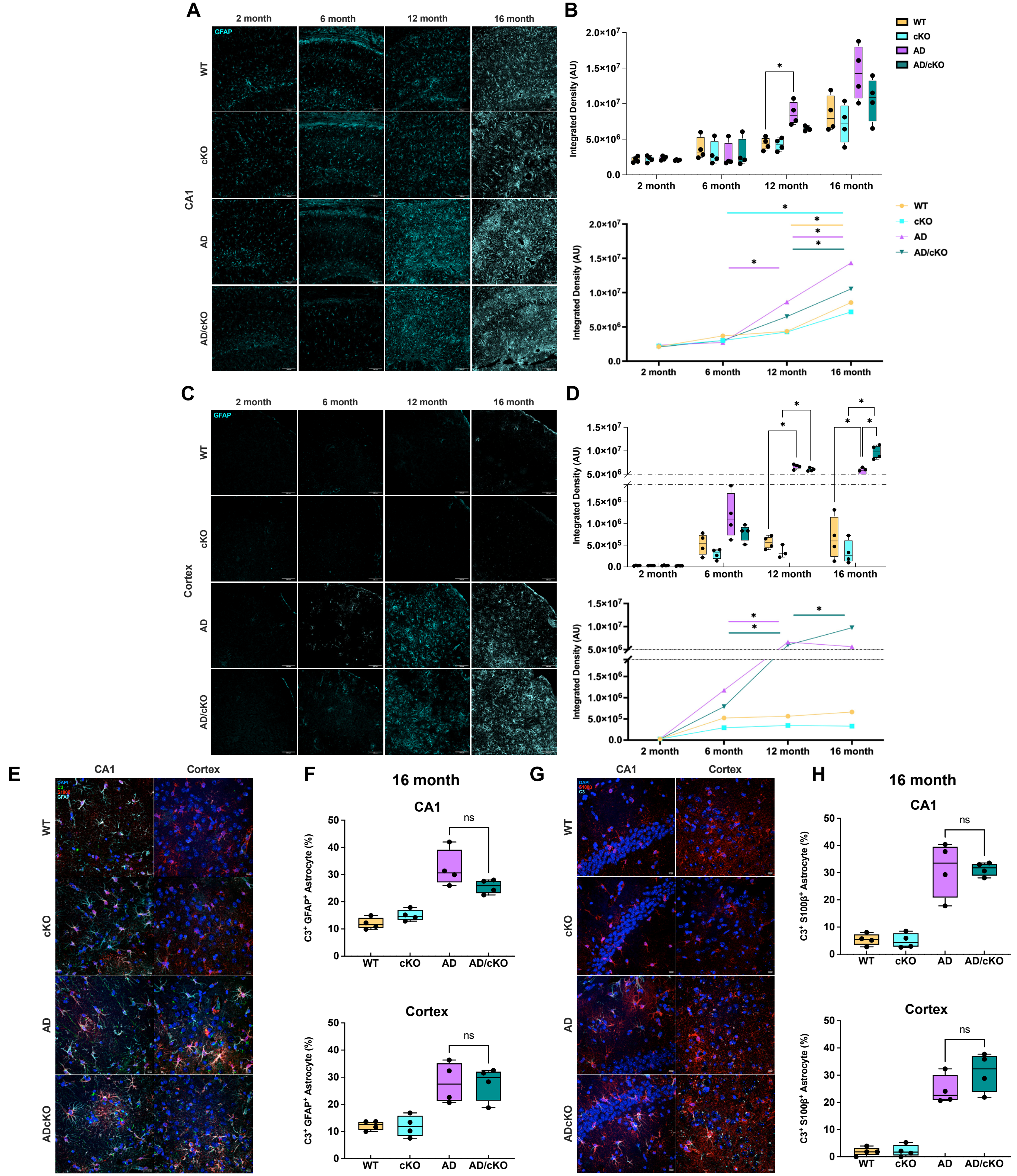


**Supplementary Figure 6. Analysis of astrocytic activation by GFAP expression and quantification of toxic astrocytes. (A)** Representative 20X confocal images of GFAP stained cells in the CA1 of 2-, 6-, 12- and 16-months old WT, cKO, AD and AD/cKO. **(B)** The graphs show GFAP+ profile density (AU) in the FOV of 3 sections in CA1 of 2-, 6-, 12- and 16-months old WT, cKO, AD and AD/cKO (*n*=four), **P*<0.05. Line plot shows the temporal course of GFAP+ profile density (AU) increases in the CA1 **P*<0.05. **(C)** Representative 20X confocal images of GFAP stained cells in the cortex of 2-, 6-, 12- and 16-months old WT, cKO, AD and AD/cKO. **(D)** The graphs show GFAP+ profile density (AU) in the FOV of 3 sections in cortex of 2-, 6-, 12- and 16-months old WT, cKO, AD and AD/cKO (*n*=four), * *P*<0.05. Line plot shows the temporal course of GFAP+ profile density (AU) increases in the cortex * *P*<0.05. **(E)** Representative 40X confocal images of GFAP (cyan) and C3(green) S100β (red) stained CA1 and cortex of 16 months old AD and AD/cKO. **(F)** The graphs show C3+/GFAP+ astrocytes in the FOV of 3 sections imaged at 20X in CA1 and cortex of 16 months old WT, cKO, AD and AD/cKO (*n*=four), ns= non-significant. **(G)** Representative 40X confocal images of C3 (cyan) and S100β (red) stained CA1 and cortex 16 months old AD and AD/cKO. **(H)** The graphs show C3+/S100β+ astrocytes in the FOV of 3 sections imaged at 20X in CA1 and cortex of 16 months old WT, cKO, AD and AD/cKO (*n*=four), ns= non-significant.

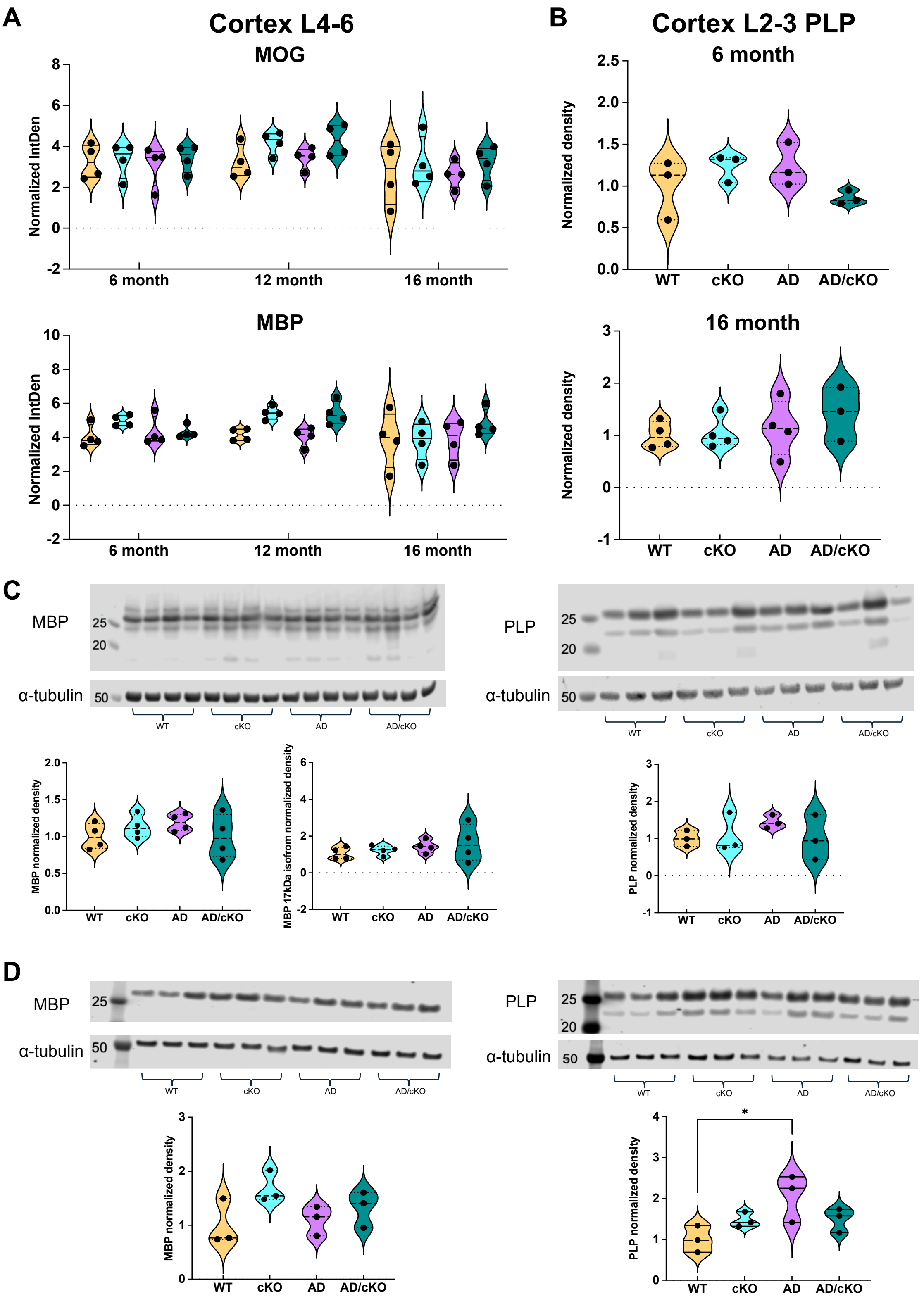


**Supplementary Figure 7. Quantification of MOG, MBP in cortical L4-6, PLP in cortical L2-3 and WB analysis of MBP and PLP proteins in the hippocampus and cortex.** **(A)** Graphs show Normalized IntDen of MOG and MBP in cortex L4-6. There are no statistically significant differences between AD and AD/cKO vs WT and cKO. **(B)** Graphs show PLP normalized intensity in cortex L2-3 at 6 and 16 months of age. There are no statistically significant differences between AD and AD/cKO vs WT and cKO. **(C)** WB of MBP and PLP in 16 months old WT, cKO, AD and AD/cKO hippocampus (HC). **(D)** WB of MBP and PLP in 16 months old WT, cKO, AD and AD/cKO cortex lysates. Graphs show quantification of total MBP, 17Kda band and PLP/DM20. There are no statistically significant differences.
