# Supplementary material for: Fus-depleted oligodendrocytes reduce neuronal damage and Alzheimer’s disease progression in the AppNL-G-F mouse
Source: Brain. Author manuscript; Available in PMC 2026 Nov 3. (PMC13730162; doi:10.1093/brain/awag234)
Supplement: Supplementary file 1 [file NIHMS2214114-supplement-supplementary_zip.zip › brain-2025-03929-File013.pdf]

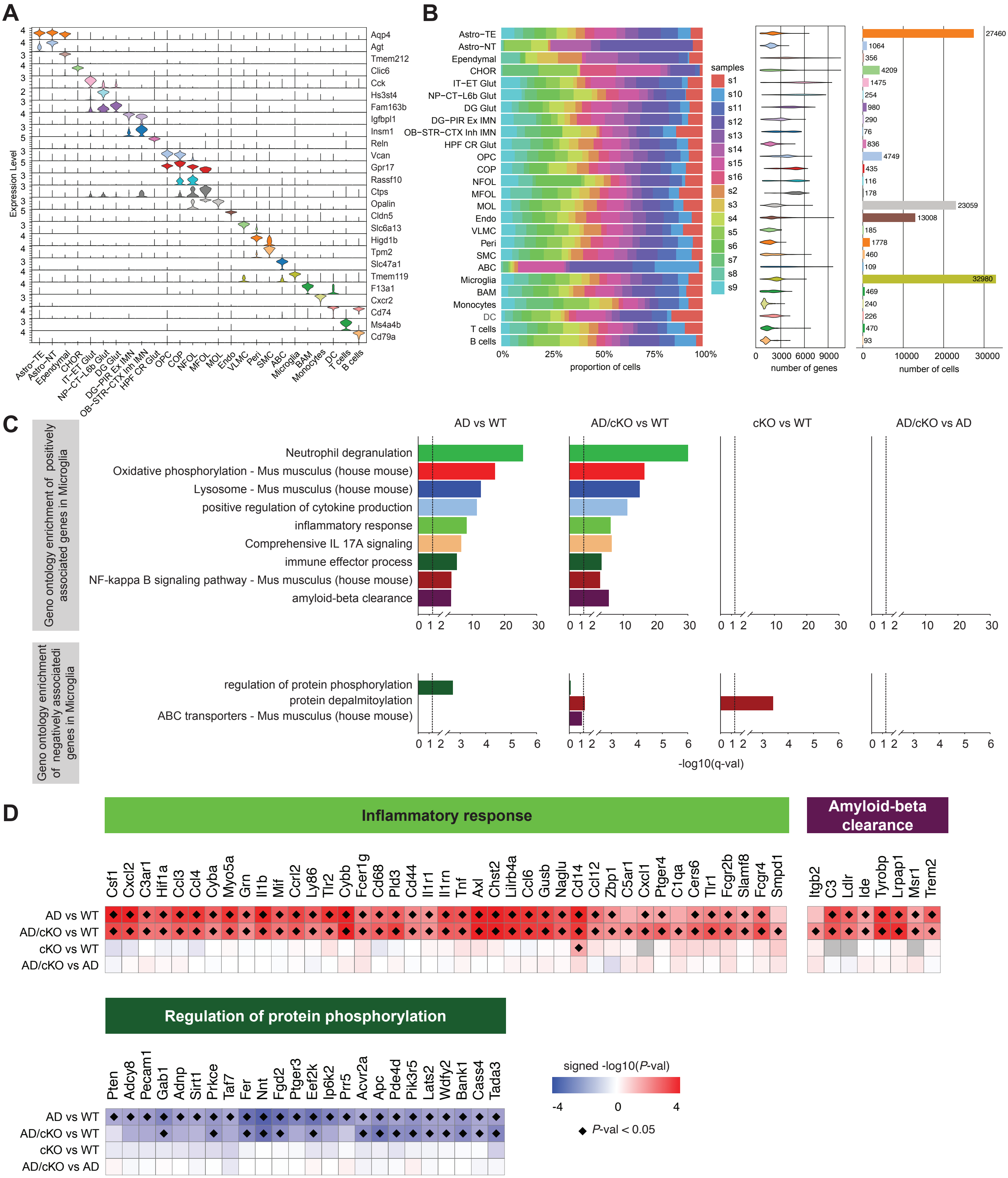

Supplementary Figure 8. Characterization of cell type populations and clusters and GO gene expression profiles in microglia. (A) Violin plot shows the expression profiles of genes used to identify the various clusters. **(B)** Heatmap and Violin plots show the number of genes expressed in each cluster. The bar graphs show the number of cells analysed in each cluster. **(C)** Gene Ontology enrichment of genes positively and negatively associated with microglia in the AD and AD/cKO. **(D)** Up-regulated genes in the AD and AD/cKO vs WT microglia are involved in inflammatory responses, while down-regulated are involved in regulation of protein phosphorylation.

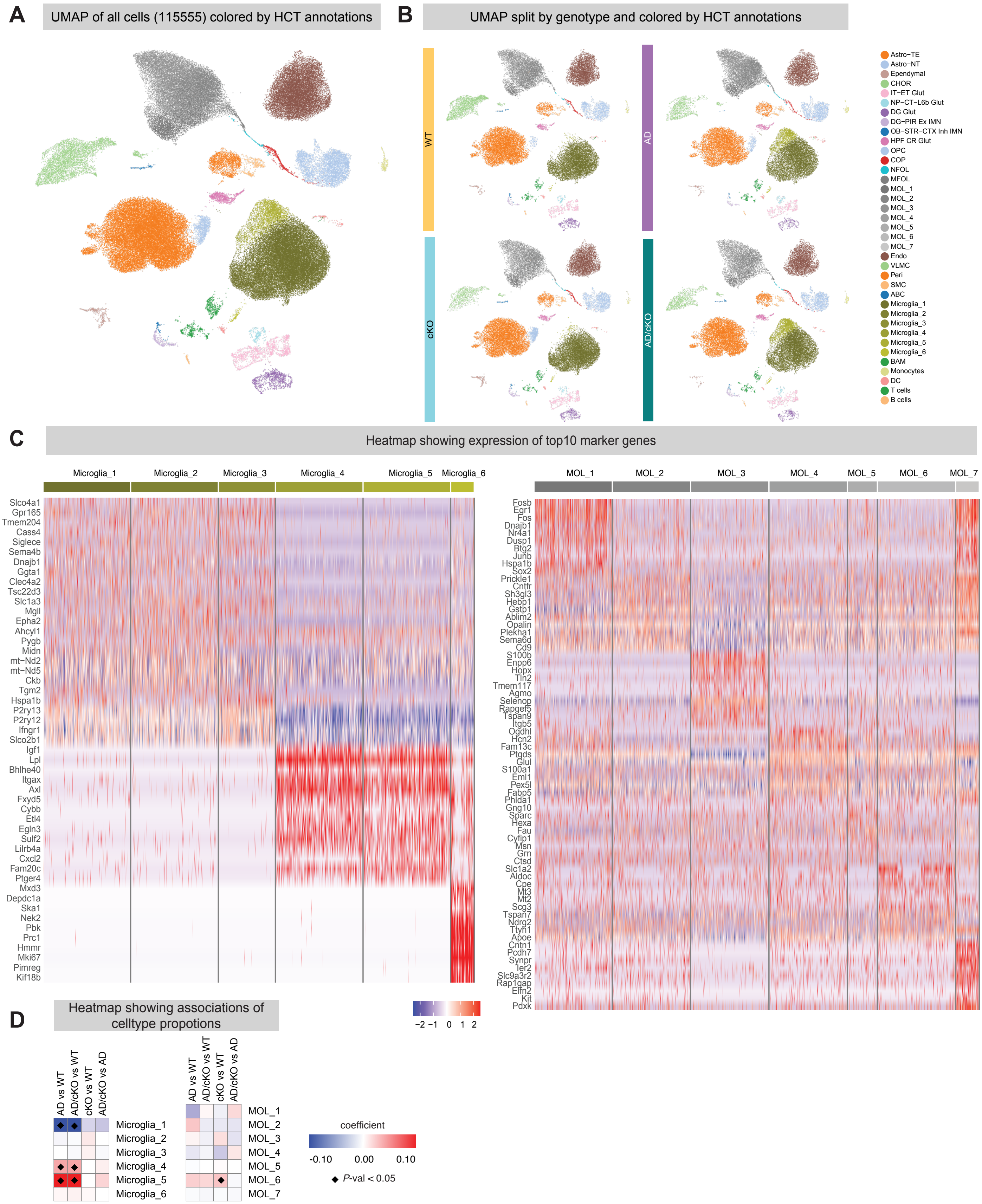

Supplementary Figure 9. Characterization of cell types and clusters per genotype. (A) Two-dimensional UMAP analysis of all cell populations identified by sc-RNA Seq according to HCT annotations. **(B)** Two-dimensional UMAP analysis of cell populations in each genotype according to HCT annotations. **(C)** Heatmap showing the 10 top expressed genes in each MOL and microglia cluster. **(D)** Heat map showing the proportional changes of cell types between AD, AD/cKO and cKO vs WT and AD/cKO vs AD for the micro clusters and the MOL clusters.
