# Supplementary material for: Fus-depleted oligodendrocytes reduce neuronal damage and Alzheimer’s disease progression in the AppNL-G-F mouse
Source: Brain. Author manuscript; Available in PMC 2026 Nov 3. (PMC13730162; doi:10.1093/brain/awag234)
Supplement: Supplementary file 1 [file NIHMS2214114-supplement-supplementary_zip.zip › brain-2025-03929-File014.pdf]

Supplementary Table 1 List of mice used in behavioural testing

Elevated Plus Maze and Y Maze	Age months	Genotype1	Genotype2	N1	N2	Attrition
Same subjects	6	AD	AD/cKO	9	14	
	6	WT	AD	9	9	
	6	WT	AD/cKO	9	14	
	6	WT	cKO	9	9	
	6	cKO	AD	9	9	
	6	cKO	AD/cKO	9	14	
Same subjects	12	AD	AD/cKO	8	14	*Loss due to death*
	12	WT	AD	11	9	
	12	WT	AD/cKO	11	14	
	12	WT	cKO	11	9	
	12	cKO	AD	9	8	*Loss due to death*
	12	cKO	AD/cKO	9	14	
New cohort	16	AD	AD/cKO	16	7	
	16	WT	AD	11	16	
	16	WT	AD/cKO	11	7	
	16	WT	cKO	11	14	
	16	cKO	AD	14	16	
	16	cKO	AD/cKO	14	7	

Supplementary Table 2 List of mice used for cholesterol quantification

Cholesterol Hippocampus and cortex*	Age months	Genotype1	Genotype2	Number1	Number2	Attrition
	2	AD	AD/cKO	8	8	
	2	WT	AD	7	8	
	2	WT	AD/cKO	7	8	
	2	WT	cKO	7	6	
	2	cKO	AD	6	8	
	2	cKO	AD/cKO	6	8	
	6	AD	AD/cKO	8	8	
	6	WT	AD	7	6	
	6	WT	AD/cKO	7	8	
	6	WT	cKO	7	6	
	6	cKO	AD	6	8	
	6	cKO	AD/cKO	6	6	
	12	AD	AD/cKO	6	5	
	12	WT	AD	4	6	
	12	WT	AD/cKO	4	5	
	12	WT	cKO	4	3	
	12	cKO	AD	3	6	
	12	cKO	AD/cKO	3	5	
	16	AD	AD/cKO	7	7	
	16	WT	AD	7	7	
	16	WT	AD/cKO	7	7	
	16	WT	cKO	7	8	
	16	cKO	AD	8	7	
	16	cKO	AD/cKO	8	7	

- Same subject's brain used for dissecting hippocampus and cortex

Supplementary Table 3 List of antibodies used in IHC and WB

Marker	Dilution	Company	Catalog number
Glial Fibrillary Acidic Protein (GFAP)	1:1000	Abcam	Ab4674
Ionized calcium-binding adaptor molecule 1 (Iba-1)	1:500	Fujifilm Wako Pure Chemical	019-19741
Myelin Basic Protein (MBP)	1:500	Abcam	Ab40390
Myelin Oligodendrocytes Glycoprotein (MOG)	1:500	Sigma-Aldrich	MAB5680
NeuN	1:800	Cell Signaling Technology	94403S
Synaptophysin	1:1000	Abcam	Ab32127
4-Hydroxynonenal (4-HNE)	1:500	Abcam	Ab46545
Triggering Receptor Expressed in Myeloid Cells (TREM2)	1:200	R&D System	AF1729
C3	1:10	Abcam	Ab11862
Proteolipid Protein (PLP)	1:2		AA3 Hybridoma
S100β	1:200	Abcam	Ab52642
β-Amyloid, Clone 6E10	1:500	BioLegend	803001
Alexa Fluor® 488 AffiniPure® Goat Anti-Mouse IgG (H+L)	1:300	Jackson ImmunoResearch Laboratories	115-545-062
Alexa Fluor® 488 AffiniPure® Goat Anti-Rat IgG (H+L)	1:300	Jackson ImmunoResearch Laboratories	112-545-003
Alexa Fluor® 568 AffiniPure® Goat Anti-Rat IgG (H+L)	1:300	Jackson ImmunoResearch Laboratories	112-575-143
Alexa Fluor® 647 AffiniPure® Goat Anti-Rat IgG (H+L)	1:300	Jackson ImmunoResearch Laboratories	112-605-003
Alexa Fluor® 594 AffiniPure® Goat Anti-Rabbit IgG (H+L)	1:300	Jackson ImmunoResearch Laboratories	111-585-003
Alexa Fluor® 647 AffiniPure® Donkey Anti-Sheep IgG (H+L)	1:300	Jackson ImmunoResearch Laboratories	713-605-147
Goat anti-Chicken IgY (H+L) Cross-Adsorbed	1:300	Invitrogen	A21103

Secondary Antibody, Alexa Fluor™ 633			
Goat anti-Rabbit IgG (H+L) Cross-Adsorbed Secondary Antibody, Alexa Fluor™ 568	1:300	Invitrogen	A11011
